# Supplementary material for: Genome-wide DNA mutations in Arabidopsis plants after multigenerational exposure to high temperatures
Source: Genome Biol. 2021 May 25;22:160. doi: 10.1186/s13059-021-02381-4 (PMC8145854; doi:10.1186/s13059-021-02381-4)
Supplement: Supplementary file 1 — Additional file 1: Table S1. Summary of whole-genome sequencing datasets of DNA samples. Table S2. Number of de novo mutations identified from MA lines and MA populations grown under Control, Heat, and Warming conditions. Table S3. List of individual sequence mutations identified in Control, Heat, and Warming MA lines and populations. Table S4. Simulated reference mutations recovered from six randomly selected MA lines. Table S5. Simulated sequence read mutations recovered from six randomly selected MA lines. Table S6. Validation of mutations detected in MA lines through conventional Sanger sequencing. Table S7. Numbers and frequencies of transitions (ts) and transversions (tv) identified in MA lines and populations grown under Control, Heat, and Warming conditions. Table S8. Comparison of mutation frequency among different genic regions calculated from Control, Heat, and Warming MA lines (A) and MA populations (B). Table S9. Whole-genome bisulfite sequencing and the numbers of methylated cytosine positions in genomic DNA samples. Table S10. Logistic regression analysis of the effects of cytosine methylation and TE regions on the likelihood of a given nucleotide being mutated in the Control, Heat, and Warming MA lines. Table S11. Genomic locations and affected bases of indels under Control, Heat, and Warming conditions. [file 13059_2021_2381_MOESM1_ESM.docx]

**Supplementary Tables**

**Table S1.** Summary of whole-genome sequencing datasets of DNA samples.

| Sample | Clean reads pairs | Clean base (G) | Q20 (%) | Q30 (%) | GC Content (%) | Average sequencing depth (X) | Coverage | Coverage at least  4X | Coverage at least 10X | Mapping |
| --- | --- | --- | --- | --- | --- | --- | --- | --- | --- | --- |
| **Ancestor** |  |  |  |  |  |  |  |  |  |  |
| G0-1 | 22,286,358 | 5.57 | 92.94 | 87.18 | 38.02 | 40.80 | 99.93% | 99.42% | 96.74% | 99.67% |
| G0-2 | 19,786,590 | 4.95 | 92.56 | 86.55 | 38.39 | 36.70 | 99.92% | 99.30% | 95.66% | 99.62% |
| G0-3 | 19,404,340 | 4.85 | 92.66 | 86.76 | 38.86 | 36.10 | 99.86% | 98.79% | 93.72% | 99.68% |
| G0-4 | 20,078,531 | 5.02 | 93.15 | 87.42 | 38.52 | 37.10 | 99.91% | 99.20% | 95.38% | 99.76% |
| G0-5 | 18,630,616 | 4.66 | 93.03 | 87.18 | 38.45 | 35.00 | 99.93% | 99.41% | 95.85% | 99.68% |
| **MA lines** |  |  |  |  |  |  |  |  |  |  |
| D10L1 | 26,849,271 | 7.88 | 97.90 | 93.95 | 37.35 | 52.93 | 99.98% | 99.96% | 99.91% | 99.23% |
| D10L2 | 24,683,771 | 7.25 | 97.75 | 93.30 | 37.30 | 51.31 | 99.98% | 99.96% | 99.92% | 99.68% |
| D10L3 | 23,327,842 | 6.85 | 97.70 | 93.20 | 37.25 | 48.93 | 99.98% | 99.96% | 99.91% | 99.63% |
| D10L4 | 28,837,474 | 8.45 | 97.60 | 92.95 | 37.20 | 59.98 | 99.98% | 99.96% | 99.94% | 99.74% |
| D10L5 | 24,561,017 | 7.21 | 97.80 | 93.80 | 37.25 | 49.53 | 99.97% | 99.95% | 99.88% | 99.33% |
| E10L1 | 25,016,326 | 7.24 | 96.95 | 91.20 | 37.25 | 50.90 | 99.97% | 99.95% | 99.91% | 99.58% |
| E10L2 | 24,426,459 | 7.17 | 97.80 | 93.85 | 37.30 | 49.60 | 99.97% | 99.95% | 99.90% | 99.57% |
| E10L3 | 26,750,100 | 7.85 | 97.75 | 93.65 | 37.30 | 53.22 | 99.97% | 99.96% | 99.91% | 99.62% |
| E10L4 | 20,113,335 | 5.89 | 97.45 | 92.60 | 37.45 | 42.16 | 99.97% | 99.95% | 99.87% | 99.77% |
| E10L5 | 25,164,799 | 7.36 | 97.45 | 92.50 | 37.30 | 52.87 | 99.98% | 99.96% | 99.92% | 99.71% |
| F10L1 | 22,104,957 | 6.48 | 97.55 | 92.70 | 37.20 | 46.81 | 99.98% | 99.96% | 99.90% | 99.74% |
| F10L2 | 16,913,539 | 4.88 | 96.50 | 90.25 | 37.35 | 35.59 | 99.98% | 99.95% | 99.66% | 99.19% |
| F10L3 | 23,125,074 | 6.77 | 97.45 | 92.55 | 37.20 | 48.75 | 99.98% | 99.96% | 99.91% | 99.79% |
| F10L4 | 28,094,709 | 8.24 | 97.80 | 93.75 | 37.30 | 56.44 | 99.97% | 99.96% | 99.92% | 99.60% |
| F10L5 | 23,878,507 | 6.91 | 96.90 | 91.00 | 37.30 | 49.28 | 99.98% | 99.96% | 99.91% | 99.58% |
| **MA populations** | |  |  |  |  |  |  |  |  |  |
| A16L1 | 20,239,038 | 5.06 | 93.18 | 87.56 | 37.52 | 37.70 | 99.96% | 99.75% | 98.21% | 99.70% |
| A16L2 | 19,930,565 | 4.98 | 93.20 | 87.52 | 38.06 | 37.20 | 99.93% | 99.48% | 96.76% | 99.78% |
| A16L3 | 29,630,044 | 7.41 | 92.96 | 87.23 | 38.42 | 53.70 | 99.91% | 99.43% | 97.73% | 99.59% |
| A16L4 | 24,834,606 | 6.21 | 94.06 | 88.95 | 37.33 | 46.00 | 99.96% | 99.79% | 98.77% | 99.88% |
| A16L5 | 21,185,484 | 5.30 | 93.98 | 88.75 | 38.64 | 39.10 | 99.87% | 99.06% | 95.42% | 99.84% |
| B22L1 | 19,648,016 | 4.91 | 94.60 | 90.10 | 37.20 | 36.30 | 99.95% | 99.75% | 98.08% | 99.74% |
| B22L2 | 19,674,015 | 4.92 | 94.71 | 90.15 | 37.14 | 36.90 | 99.96% | 99.83% | 98.56% | 99.74% |
| B22L3 | 19,657,466 | 4.91 | 94.11 | 89.14 | 38.39 | 36.90 | 99.78% | 98.60% | 94.36% | 99.85% |
| B22L4 | 17,711,274 | 4.43 | 94.19 | 89.25 | 37.66 | 33.50 | 99.91% | 99.22% | 95.43% | 99.76% |
| B22L5 | 23,748,730 | 5.94 | 94.10 | 89.04 | 38.03 | 43.60 | 99.92% | 99.41% | 97.08% | 99.85% |
| C19L1 | 19,708,913 | 4.93 | 93.86 | 88.60 | 37.34 | 37.10 | 99.92% | 99.50% | 97.43% | 99.85% |
| C19L2 | 17,825,506 | 4.46 | 93.64 | 88.22 | 37.50 | 33.90 | 99.88% | 99.23% | 96.16% | 99.84% |
| C19L3 | 18,576,698 | 4.64 | 93.59 | 88.16 | 37.66 | 35.00 | 99.93% | 99.43% | 96.35% | 99.62% |
| C19L4 | 18,238,555 | 4.56 | 93.42 | 87.88 | 37.34 | 34.40 | 99.94% | 99.64% | 97.44% | 99.84% |
| C19L5 | 21,858,506 | 5.47 | 93.91 | 88.69 | 37.77 | 41.00 | 99.94% | 99.61% | 97.77% | 99.85% |

**Table S2.** Number of de novo mutations identified from MA lines and MA populations grown under Control, Heat, and Warming conditions.

| Category | Sample | SNV | indel  (Insertion, Deletion)^a^ | Total number of mutation |
| --- | --- | --- | --- | --- |
| **MA lines** | D10L1 | 2 | 2 (0, 2) | 4 |
| Control | D10L2 | 8 | 2 (2, 0) | 10 |
|  | D10L3 | 8 | 3 (2, 1) | 10 |
|  | D10L4 | 7 | 0 (0, 0) | 7 |
|  | D10L5 | 6 | 1 (0, 1) | 7 |
|  | Mean | 6.2 | 1.6 (0.8/0.8) | 7.8 |
| Heat | E10L1 | 15 | 5 (1, 4) | 20 |
|  | E10L2 | 14 | 5 (1, 4) | 19 |
|  | E10L3 | 16 | 6 (1, 5) | 22 |
|  | E10L4 | 14 | 5 (0, 5) | 19 |
|  | E10L5 | 10 | 8 (1, 7) | 18 |
|  | Mean | 14 | 5.8 (0.8/5.0) | 19.6 |
| Warming | F10L1 | 9 | 3 (2, 1) | 12 |
|  | F10L2 | 11 | 4 (2, 3) | 15 |
|  | F10L3 | 12 | 7 (0, 5) | 19 |
|  | F10L4 | 13 | 4 (0, 4) | 17 |
|  | F10L5 | 9 | 2 (0, 2) | 11 |
|  | Mean | 10.8 | 4.0 (0.6/3.4) | 14.8 |
| **MA populations** |  |  |  |  |
| Control | A16L1 | 9 | 2 (1, 1) | 11 |
|  | A16L2 | 17 | 2 (1, 1) | 19 |
|  | A16L3 | 12 | 2 (2, 0) | 14 |
|  | A16L4 | 12 | 3 (2, 1) | 15 |
|  | A16L5 | 10 | 1 (1, 0) | 11 |
|  | Mean | 12.0 | 2.0 (1.4/0.6) | 14.0 |
| Heat | B22L1 | 26 | 8 (1, 7) | 34 |
|  | B22L2 | 25 | 13 (3, 10) | 38 |
|  | B22L3 | 23 | 8 (1, 7) | 31 |
|  | B22L4 | 24 | 11 (2, 9) | 35 |
|  | B22L5 | 32 | 13 (4, 9) | 45 |
|  | Mean | 31.8 | 10.6 (2.2/8.4) | 36.6 |
| Warming | C19L1 | 14 | 7 (2, 5) | 21 |
|  | C19L2 | 21 | 9 (3, 6) | 30 |
|  | C19L3 | 15 | 5 (1, 4) | 20 |
|  | C19L4 | 20 | 6 (3, 3) | 26 |
|  | C19L5 | 18 | 8 (4, 4) | 26 |
|  | Mean | 17.6 | 7.0 (2.6/4.4) | 24.6 |

^a^Indel ≤ 50bp.

**Table S3** List of individual sequence mutations identified in Control, Heat, and Warming MA lines and populations.

| Sample | Position | Mutation | Annotation | Gene ID |
| --- | --- | --- | --- | --- |
| D11L1 | Chr1_28180441 | A->G | exonic:synonymous | AT1G75050 |
| D11L1 | Chr1_30045923 | G->A | UTR3 | AT1G79870 |
| D11L1 | Chr2_9481938 | deletion(6): TTGAGT | ncRNA:lncRNA |  |
| D11L1 | Chr3_3299184 | deletion(1): A | upstream |  |
| D11L1 | Chr5_21796347 | G->A | upstream;downstream |  |
| D11L2 | Chr1_8500098 | insertion(1): A | upstream;downstream |  |
| D11L2 | Chr1_10021083 | T->G | downstream |  |
| D11L2 | Chr1_12741653 | C->T | intergenic |  |
| D11L2 | Chr1_20755080 | G->A | exonic:synonymous | AT1G55560 |
| D11L2 | Chr3_6651225 | insertion(2): CT | intergenic |  |
| D11L2 | Chr3_12090975 | G->A | intergenic |  |
| D11L2 | Chr3_13795890 | A->G | intergenic |  |
| D11L2 | Chr3_20040388 | A->G | intronic | AT3G54110 |
| D11L2 | Chr5_8812541 | G->A | intergenic |  |
| D11L2 | Chr5_22379192 | T->A | intronic | AT5G55132 |
| D11L3 | Chr1_5277948 | A->G | upstream;downstream |  |
| D11L3 | Chr1_15903567 | T->C | intergenic |  |
| D11L3 | Chr2_17323097 | insertion(1): T | downstream |  |
| D11L3 | Chr3_9444815 | G->T | transposable element | AT3TE39430 |
| D11L3 | Chr4_388431 | insertion(3): CTT | exonic:nonframeshift | AT4G00905 |
| D11L3 | Chr4_8020162 | A->G | upstream |  |
| D11L3 | Chr5_8088478 | deletion(1): T | transposable element | AT5TE29250 |
| D11L3 | Chr5_13481434 | G->C | intronic | AT5G35210 |
| D11L3 | Chr5_19141355 | G->A | intronic | AT5G47130 |
| D11L4 | Chr1_13264193 | C->T | intergenic |  |
| D11L4 | Chr2_2740878 | C->T | transposable element | AT2TE12115 |
| D11L4 | Chr3_2550672 | A->T | intronic | AT3G07980 |
| D11L4 | Chr3_4835343 | A->T | intergenic |  |
| D11L4 | Chr3_13858270 | T->C | intergenic |  |
| D11L4 | Chr5_3805033 | T->C | exonic:synonymous | AT5G11800 |
| D11L4 | Chr5_12023299 | A->C | intergenic |  |
| D11L4 | Chr5_16014724 | G->C | intergenic |  |
| D11L5 | Chr1_7080936 | C->T | intronic | AT1G20410 |
| D11L5 | Chr1_12024468 | T->C | exonic:nonsynonymous | AT1G33140 |
| D11L5 | Chr1_15563567 | G->A | intergenic |  |
| D11L5 | Chr2_5891163 | G->A | transposable element | AT2TE23965 |
| D11L5 | Chr2_13702918 | deletion(1): T | intergenic |  |
| D11L5 | Chr2_19697643 | G->A | intergenic |  |
| D11L5 | Chr5_15272931 | C->T | downstream |  |
| E11L1 | Chr1_4299919 | T->A | downstream |  |
| E11L1 | Chr1_17440330 | C->T | intergenic |  |
| E11L1 | Chr1_21858117 | C->T | downstream |  |
| E11L1 | Chr2_509829 | A->G | UTR3 | AT2G02070 |
| E11L1 | Chr2_3232467 | C->G | intergenic |  |
| E11L1 | Chr2_4041454 | C->T | transposable element | AT2TE17110 |
| E11L1 | Chr2_12003173 | deletion(1): T | downstream |  |
| E11L1 | Chr3_4368321 | C->T | exonic:nonsynonymous | AT3G13430 |
| E11L1 | Chr3_17912372 | deletion(1): A | downstream |  |
| E11L1 | Chr4_112973 | G->A | exonic:nonsynonymous | AT4G00250 |
| E11L1 | Chr4_4060978 | G->A | intergenic |  |
| E11L1 | Chr4_10783884 | A->G | exonic:synonymous | AT4G19870 |
| E11L1 | Chr4_11368743 | C->T | upstream |  |
| E11L1 | Chr4_14450247 | C->T | exonic:nonsynonymous | AT4G29350 |
| E11L1 | Chr4_17786593 | deletion(1): A | exonic:frameshift deletion | AT4G37820 |
| E11L1 | Chr5_8721045 | C->A | transposable element | AT5TE31545 |
| E11L1 | Chr5_10735764 | G->A | intergenic |  |
| E11L1 | Chr5_13526118 | insertion(1): T | upstream;downstream |  |
| E11L1 | Chr5_15646457 | G->A | transposable element | AT5TE56585 |
| E11L1 | Chr5_18040165 | deletion(1): A | upstream |  |
| E11L2 | Chr1_1592311 | A->T | intergenic |  |
| E11L2 | Chr1_2481570 | T->C | intronic | AT1G07990 |
| E11L2 | Chr1_7312386 | T->C | intergenic |  |
| E11L2 | Chr1_16140730 | deletion(1): T | intronic | AT1G43000 |
| E11L2 | Chr1_17863094 | G->A | upstream |  |
| E11L2 | Chr1_18097747 | deletion(4): TTTC | upstream |  |
| E11L2 | Chr3_9739441 | G->A | intergenic |  |
| E11L2 | Chr3_13783756 | T->A | intergenic |  |
| E11L2 | Chr3_14249144 | C->G | transposable element | AT3TE58580 |
| E11L2 | Chr3_17451284 | G->C | exonic:nonsynonymous | AT3G47360 |
| E11L2 | Chr4_3507964 | insertion(15): TTGATGTTATAGAGG | transposable element | AT4TE15820 |
| E11L2 | Chr4_9872289 | C->A | exonic:nonsynonymous | AT4G17760 |
| E11L2 | Chr4_11591112 | deletion(1): A | downstream |  |
| E11L2 | Chr5_8320799 | G->T | upstream;downstream |  |
| E11L2 | Chr5_8320806 | T->C | upstream;downstream |  |
| E11L2 | Chr5_8320843 | A->C | upstream;downstream |  |
| E11L2 | Chr5_13744565 | C->T | exonic:synonymous | AT5G35560 |
| E11L2 | Chr5_13942107 | C->T | intergenic |  |
| E11L2 | Chr5_11310914 | deletion(1): T | intergenic |  |
| E11L3 | Chr1_5841059 | A->T | intronic | AT1G17080 |
| E11L3 | Chr1_11957865 | deletion(1): T | upstream;downstream |  |
| E11L3 | Chr1_13301299 | G->A | intergenic |  |
| E11L3 | Chr1_24553233 | T->A | UTR5 | AT1G65960 |
| E11L3 | Chr2_1761184 | deletion(3): AGA | transposable element | AT2TE08135 |
| E11L3 | Chr2_4995013 | T->A | transposable element | AT2TE20450 |
| E11L3 | Chr2_5037964 | T->A | transposable element | AT2TE20710 |
| E11L3 | Chr2_5414127 | C->T | transposable element | AT2TE21925 |
| E11L3 | Chr2_5536209 | A->G | downstream |  |
| E11L3 | Chr2_8060293 | insertion(1): G | exonic:frameshift | AT2G18560 |
| E11L3 | Chr2_16333363 | T->C | UTR3 | AT2G39140 |
| E11L3 | Chr2_16405434 | G->A | exonic:synonymous | AT2G39280 |
| E11L3 | Chr3_16830078 | T->A | exonic:nonsynonymous | AT3G45800 |
| E11L3 | Chr3_18945978 | T->A | exonic:nonsynonymous | AT3G51000 |
| E11L3 | Chr3_19139288 | deletion(1): T | UTR5 | AT3G51600 |
| E11L3 | Chr3_20098935 | G->A | exonic:nonsynonymous | AT3G54280 |
| E11L3 | Chr4_5555374 | A->C | UTR3 | AT4G08691 |
| E11L3 | Chr4_18096196 | deletion(2): GA | UTR5 | AT4G38760 |
| E11L3 | Chr5_1072079 | C->T | exonic:nonsynonymous | AT5G03970 |
| E11L3 | Chr5_16154747 | C->A | exonic:synonymous | AT5G40380 |
| E11L3 | Chr5_19774483 | deletion(1): G | exonic:frameshift | AT5G48770 |
| E11L3 | Chr5_26949851 | C->T | upstream |  |
| E11L4 | Chr1_752141 | T->C | upstream;downstream |  |
| E11L4 | Chr1_11509302 | G->T | upstream;downstream |  |
| E11L4 | Chr1_16038306 | C->T | intronic | AT1G42615 |
| E11L4 | Chr1_18941043 | deletion(1): A | intronic | AT1G51130 |
| E11L4 | Chr1_22224924 | G->A | upstream |  |
| E11L4 | Chr1_24896437 | G->A | exonic:nonsynonymous | AT1G66750 |
| E11L4 | Chr1_26847185 | deletion(1): A | intronic | AT1G71220 |
| E11L4 | Chr2_1655747 | deletion(2): TA | upstream |  |
| E11L4 | Chr2_5198118 | A->T | intergenic |  |
| E11L4 | Chr2_8680343 | C->T | intronic | AT2G20100 |
| E11L4 | Chr3_10719893 | deletion(1): T | intergenic |  |
| E11L4 | Chr3_12717669 | C->T | intergenic |  |
| E11L4 | Chr3_13525199 | deletion(1): A | downstream |  |
| E11L4 | Chr3_13693107 | G->T | intergenic |  |
| E11L4 | Chr4_963036 | C->T | exonic:nonsynonymous | AT4G02180 |
| E11L4 | Chr4_3562787 | G->C | intergenic |  |
| E11L4 | Chr5_8320803 | G->A | upstream;downstream |  |
| E11L4 | Chr5_12203220 | T->C | transposable element | AT5TE43385 |
| E11L4 | Chr5_12262232 | T->A | intergenic |  |
| E11L5 | Chr1_1933382 | deletion(1): A | UTR3 | AT1G06340 |
| E11L5 | Chr1_13297903 | G->A | intergenic |  |
| E11L5 | Chr1_18132357 | G->C | upstream |  |
| E11L5 | Chr1_21128436 | T->A | upstream |  |
| E11L5 | Chr2_3051713 | T->G | intronic | AT2G07360 |
| E11L5 | Chr2_13868472 | G->A | intronic | AT2G32700 |
| E11L5 | Chr3_234616 | deletion(1): T | intronic | AT3G01610 |
| E11L5 | Chr3_9785615 | G->A | transposable element | AT3TE40735 |
| E11L5 | Chr3_12752365 | G->A | transposable element | AT3TE52445 |
| E11L5 | Chr4_1507947 | insertion(2): AT | intergenic |  |
| E11L5 | Chr4_9377511 | A->G | exonic:nonsynonymous | AT4G16660 |
| E11L5 | Chr4_12102010 | T->G | upstream;downstream |  |
| E11L5 | Chr4_14602866 | deletion(1): G | exonic:stopgain | AT4G29850 |
| E11L5 | Chr5_1383661 | deletion(1): T | upstream |  |
| E11L5 | Chr5_7909462 | deletion(2): GA | UTR5 | AT5G23450 |
| E11L5 | Chr5_12089326 | deletion(1): T | transposable element | AT5TE43130 |
| E11L5 | Chr5_20729723 | A->G | exonic:nonsynonymous | AT5G50950 |
| E11L5 | Chr5_21660951 | deletion(1): A | upstream |  |
| F11L1 | Chr1_6677325 | insertion(1): A | intronic | AT1G19310 |
| F11L1 | Chr1_9094442 | C->A | intergenic |  |
| F11L1 | Chr1_12130437 | A->G | UTR5 | AT1G33440 |
| F11L1 | Chr2_3966820 | C->T | intergenic |  |
| F11L1 | Chr3_16939107 | G->A | intronic | AT3G46120 |
| F11L1 | Chr3_20175685 | A->G | intronic | AT3G54500 |
| F11L1 | Chr4_3678327 | G->A | intergenic |  |
| F11L1 | Chr4_6635396 | C->T | exonic:nonsynonymous | AT4G10780 |
| F11L1 | Chr5_5229726 | insertion(3): CTT | exonic:nonframeshift | AT5G16020 |
| F11L1 | Chr5_5933484 | A->T | downstream |  |
| F11L1 | Chr5_14023478 | G->A | intergenic |  |
| F11L1 | Chr5_24779066 | deletion(2): TT | UTR3 | AT5G61650 |
| F11L2 | Chr1_26011710 | deletion(1): C | intergenic |  |
| F11L2 | Chr1_27740161 | C->T | splicing | AT1G73760 |
| F11L2 | Chr2_2800305 | C->T | upstream |  |
| F11L2 | Chr2_3225533 | C->T | transposable element | AT2TE13950 |
| F11L2 | Chr2_13971245 | G->A | downstream |  |
| F11L2 | Chr3_8212836 | deletion(22): GTTTGAAAACAGCTATCGTGCT | transposable element | AT3TE34445 |
| F11L2 | Chr3_11094358 | insertion(1): | transposable element | AT3TE46155 |
| F11L2 | Chr3_11638846 | C->T | intergenic |  |
| F11L2 | Chr4_2844088 | G->A | intergenic |  |
| F11L2 | Chr4_3151045 | C->T | intergenic |  |
| F11L2 | Chr4_7341497 | C->A | ncRNA:tRNA |  |
| F11L2 | Chr4_1949803 | G->A | intergenic |  |
| F11L2 | Chr4_18055621 | deletion(12): CATACATACATA | downstream |  |
| F11L2 | Chr5_19427454 | T->C | intergenic |  |
| F11L2 | Chr5_20838995 | T->A | UTR5 | AT5G51270 |
| F11L3 | Chr1_5499962 | G->A | exonic:synonymous | AT1G16020 |
| F11L3 | Chr1_14056770 | T->C | transposable element | AT1TE46180 |
| F11L3 | Chr1_15432695 | C->T | exonic:nonsynonymous | AT1G40390 |
| F11L3 | Chr1_15607028 | deletion(1): T | intronic | AT1G41830 |
| F11L3 | Chr2_1135101 | C->T | intergenic |  |
| F11L3 | Chr2_6049268 | G->T | transposable element | AT2TE24635 |
| F11L3 | Chr2_11097454 | deletion(1): A | transposable element | AT2TE47865 |
| F11L3 | Chr2_12387869 | deletion(1): C | downstream |  |
| F11L3 | Chr2_16919679 | deletion(1): G | UTR3 | AT2G40510 |
| F11L3 | Chr3_5942504 | A->T | exonic:nonsynonymous | AT3G17360 |
| F11L3 | Chr3_15666533 | T->A | upstream |  |
| F11L3 | Chr3_16077875 | deletion(22): AAACATTGAATAATGTTTACCT | transposable element | AT3TE65055 |
| F11L3 | Chr3_16672443 | C->T | upstream |  |
| F11L3 | Chr4_5545183 | deletion(1): T | upstream |  |
| F11L3 | Chr4_812950 | deletion(1): T | upstream |  |
| F11L3 | Chr4_13277334 | G->T | downstream |  |
| F11L3 | Chr5_3275501 | T->A | intronic | AT5G10420 |
| F11L3 | Chr5_4812341 | C->A | intergenic |  |
| F11L3 | Chr5_17598182 | C->T | transposable element | AT5TE63610 |
| F11L4 | Chr1_1560460 | A->G | upstream;downstream |  |
| F11L4 | Chr1_1576361 | G->T | upstream |  |
| F11L4 | Chr1_10307932 | deletion(9): TAGTAGTAG | exonic:nonframeshift | AT1G29460 |
| F11L4 | Chr1_20544609 | G->C | exonic:nonsynonymous | AT1G55050 |
| F11L4 | Chr1_29665186 | G->A | UTR5 | AT1G78910 |
| F11L4 | Chr2_945764 | deletion(18): GATGAGAAGAATCTCAAG | exonic:nonframeshift | AT2G03140 |
| F11L4 | Chr2_1998090 | C->T | transposable element | AT2TE09205 |
| F11L4 | Chr2_17437659 | A->G | exonic:nonsynonymous | AT2G41800 |
| F11L4 | Chr3_5779905 | G->A | downstream |  |
| F11L4 | Chr3_10500038 | T->A | exonic:synonymous | AT3G28170 |
| F11L4 | Chr3_12174170 | C->T | transposable element | AT3TE50550 |
| F11L4 | Chr3_18747765 | C->T | upstream |  |
| F11L4 | Chr4_11446995 | A->T | exonic:nonsynonymous | AT4G21510 |
| F11L4 | Chr5_2165591 | A->T | exonic:nonsynonymous | AT5G06970 |
| F11L4 | Chr5_10083527 | deletion(1): T | upstream |  |
| F11L4 | Chr5_13772087 | deletion(3): AAT | upstream |  |
| F11L4 | Chr5_17063924 | A->G | exonic:nonsynonymous | AT5G42610 |
| F11L5 | Chr1_12299829 | T->A | intergenic |  |
| F11L5 | Chr1_12299830 | A->T | intergenic |  |
| F11L5 | Chr1_21480740 | deletion(3): TCT | exonic:frameshift | AT1G58050 |
| F11L5 | Chr1_25387621 | A->C | exonic:nonsynonymous | AT1G67720 |
| F11L5 | Chr1_25718399 | A->G | intergenic |  |
| F11L5 | Chr2_602407 | C->T | upstream |  |
| F11L5 | Chr2_4554916 | G->A | intergenic |  |
| F11L5 | Chr2_13881660 | deletion(2): GA | exonic:frameshift | AT2G32730 |
| F11L5 | Chr3_4333069 | C->T | intronic | AT3G13340 |
| F11L5 | Chr3_16705392 | C->G | downstream |  |
| F11L5 | Chr5_2965228 | A->T | exonic:synonymous | AT5G09550 |
| A17L1 | Chr1_7961216 | G->A | intergenic |  |
| A17L1 | Chr2_4736385 | G->A | transposable element | AT2TE19615 |
| A17L1 | Chr2_9107095 | T->C | intronic | AT2G21260 |
| A17L1 | Chr3_5640201 | G->A | intronic | AT3G16565 |
| A17L1 | Chr4_554025 | C->T | UTR3 | AT4G01335 |
| A17L1 | Chr5_2304309 | A->T | intergenic |  |
| A17L1 | Chr5_11366010 | C->T | exonic:synonymous | AT5G30341 |
| A17L1 | Chr5_18598079 | A->T | upstream |  |
| A17L1 | Chr4_10705602 | deletion (1): T | downstream |  |
| A17L1 | Chr4_14381494 | insertion(1): A | intronic | AT4G29160 |
| A17L1 | Chr4_16826377 | T->G | downstream |  |
| A17L2 | Chr1_7961216 | G->A | intergenic |  |
| A17L2 | Chr1_9426606 | T->C | UTR3 | AT1G27130 |
| A17L2 | Chr1_16723818 | C->T | downstream |  |
| A17L2 | Chr2_4736385 | G->A | transposable element | AT2TE19615 |
| A17L2 | Chr2_6158323 | C->G | transposable element | AT2TE25020 |
| A17L2 | Chr2_9107095 | T->C | intronic | AT2G21260 |
| A17L2 | Chr3_5640201 | G->A | intronic | AT3G16565 |
| A17L2 | Chr3_16525472 | T->G | exonic:synonymous | AT3G45140 |
| A17L2 | Chr3_17433511 | G->A | transposable element | AT3TE70635 |
| A17L2 | Chr3_17654749 | G->A | exonic:nonsynonymous | AT3G47850 |
| A17L2 | Chr4_999926 | T->C | downstream |  |
| A17L2 | Chr4_4270702 | C->T | intergenic |  |
| A17L2 | Chr4_14381494 | insertion(1): A | intronic | AT4G29160 |
| A17L2 | Chr4_16826377 | T->G | downstream |  |
| A17L2 | Chr5_12160043 | G->A | intergenic |  |
| A17L2 | Chr5_13542424 | G->A | intergenic |  |
| A17L2 | Chr5_15232899 | G->A | intergenic |  |
| A17L2 | Chr5_18598079 | A->T | upstream |  |
| A17L2 | Chr5_20525981 | deletion(1): T | intronic | AT5G50400 |
| A17L3 | Chr1_7961216 | G->A | intergenic |  |
| A17L3 | Chr1_9426606 | T->C | UTR3 | AT1G27130 |
| A17L3 | Chr1_16723818 | C->T | downstream |  |
| A17L3 | Chr1_30195910 | G->A | exonic:synonymous | AT1G80310 |
| A17L3 | Chr2_4736385 | G->A | transposable element | AT2TE19615 |
| A17L3 | Chr2_8070413 | C->T | exonic:synonymous | AT2G18590 |
| A17L3 | Chr2_9107095 | T->C | intronic | AT2G21260 |
| A17L3 | Chr2_9187977 | A->C | intergenic |  |
| A17L3 | Chr3_5640201 | G->A | intronic | AT3G16565 |
| A17L3 | Chr3_7976019 | insertion(1): T | exonic:frameshift | AT3G22520 |
| A17L3 | Chr4_2714052 | G->A | upstream;downstream |  |
| A17L3 | Chr4_14381494 | insertion(1): A | intronic | AT4G29160 |
| A17L3 | Chr4_16826377 | T->G | downstream |  |
| A17L3 | Chr5_18598079 | A->T | upstream |  |
| A17L4 | Chr1_3847211 | G->C | upstream |  |
| A17L4 | Chr1_7961216 | G->A | intergenic |  |
| A17L4 | Chr1_16723818 | C->T | downstream |  |
| A17L4 | Chr1_30195910 | G->A | exonic:synonymous | AT1G80310 |
| A17L4 | Chr2_2539975 | G->C | exonic:nonsynonymous | AT2G06420 |
| A17L4 | Chr2_4736385 | G->A | transposable element | AT2TE19615 |
| A17L4 | Chr2_9107095 | T->C | intronic | AT2G21260 |
| A17L4 | Chr3_3154818 | C->A | upstream |  |
| A17L4 | Chr3_5640201 | G->A | intronic | AT3G16565 |
| A17L4 | Chr3_12506064 | deletion(1): A | transposable element | AT3TE51545 |
| A17L4 | Chr3_18672292 | insertion(1): A | intronic | AT3G50350 |
| A17L4 | Chr4_3887649 | A->G | transposable element | AT4TE16990 |
| A17L4 | Chr4_14381494 | insertion(1): A | intronic | AT4G29160 |
| A17L4 | Chr4_16826377 | T->G | downstream |  |
| A17L4 | Chr5_18598079 | A->T | upstream |  |
| A17L5 | Chr1_7961216 | G->A | intergenic |  |
| A17L5 | Chr1_16681470 | G->A | splicing | AT1G43970 |
| A17L5 | Chr1_16723818 | C->T | downstream |  |
| A17L5 | Chr2_4736385 | G->A | transposable element | AT2TE19615 |
| A17L5 | Chr2_5785483 | C->T | transposable element | AT2TE23510 |
| A17L5 | Chr2_9107095 | T->C | intronic | AT2G21260 |
| A17L5 | Chr3_5640201 | G->A | intronic | AT3G16565 |
| A17L5 | Chr3_14421935 | G->A | intergenic |  |
| A17L5 | Chr4_14381494 | insertion(1): A | intronic | AT4G29160 |
| A17L5 | Chr4_16826377 | T->G | downstream |  |
| A17L5 | Chr5_18598079 | A->T | upstream |  |
| B23L1 | Chr1_12376491 | A->G | downstream |  |
| B23L1 | Chr1_12651414 | C->T | intronic | AT1G34550 |
| B23L1 | Chr1_13549753 | G->T | intronic | AT1G36180 |
| B23L1 | Chr1_14687243 | C->T | intergenic |  |
| B23L1 | Chr1_16389418 | G->A | transposable element gene | AT1G43502 |
| B23L1 | Chr1_17296298 | deletion(1): A | intergenic |  |
| B23L1 | Chr1_21055169 | deletion(1): T | intergenic |  |
| B23L1 | Chr1_23153513 | A->G | exonic:nonsynonymous | AT1G62540 |
| B23L1 | Chr1_26435332 | G->A | upstream |  |
| B23L1 | Chr2_396945 | T->G | UTR3 | AT2G01890 |
| B23L1 | Chr3_2609728 | deletion(1): C | intronic | AT3G08590 |
| B23L1 | Chr3_5568481 | C->A | UTR3 | AT3G16400 |
| B23L1 | Chr3_12426326 | deletion(1): A | transposable element | AT3TE51350 |
| B23L1 | Chr3_12806614 | C->T | intergenic |  |
| B23L1 | Chr3_14258703 | A->G | transposable element | AT3TE58605 |
| B23L1 | Chr3_15732338 | C->T | intergenic |  |
| B23L1 | Chr3_16708447 | C->T | upstream |  |
| B23L1 | Chr3_20062358 | T->A | exonic:nonsynonymous | AT3G54190 |
| B23L1 | Chr4_4418072 | C->T | intergenic |  |
| B23L1 | Chr4_4462702 | T->C | transposable element | AT4TE18845 |
| B23L1 | Chr4_5134021 | T->A | intergenic |  |
| B23L1 | Chr4_5896901 | C->T | intergenic |  |
| B23L1 | Chr4_6081717 | C->T | transposable element | AT4TE25635 |
| B23L1 | Chr4_8844639 | G->C | intronic | AT4G15470 |
| B23L1 | Chr4_17051455 | T->A | upstream;downstream |  |
| B23L1 | Chr5_7356477 | deletion(1): T | intronic | AT5G22200 |
| B23L1 | Chr5_9872614 | C->T | upstream |  |
| B23L1 | Chr5_10692053 | C->T | transposable element | AT5TE38935 |
| B23L1 | Chr5_12499229 | A->T | pseudogene | AT5G33251 |
| B23L1 | Chr5_12646742 | C->T | transposable element | AT5TE44770 |
| B23L1 | Chr5_13976747 | deletion(1): T | transposable element | AT5TE49800 |
| B23L1 | Chr5_14563554 | T->G | upstream;downstream |  |
| B23L1 | Chr5_15578649 | deletion(1): A | upstream |  |
| B23L1 | Chr5_23370350 | insertion(2): TT | intergenic |  |
| B23L2 | Chr1_12376491 | A->G | downstream |  |
| B23L2 | Chr1_12651414 | C->T | intronic | AT1G34550 |
| B23L2 | Chr1_12736204 | insertion(1): A | UTR3 | AT1G34750 |
| B23L2 | Chr1_13549753 | G->T | intronic | AT1G36180 |
| B23L2 | Chr1_14687243 | C->T | intergenic |  |
| B23L2 | Chr1_16389418 | G->A | transposable element gene | AT1G43502 |
| B23L2 | Chr1_17211131 | deletion(1): A | intergenic |  |
| B23L2 | Chr1_17296298 | deletion(1): A | intergenic |  |
| B23L2 | Chr1_21055169 | deletion(1): T | intergenic |  |
| B23L2 | Chr1_26435332 | G->A | upstream |  |
| B23L2 | Chr2_396945 | T->G | UTR3 | AT2G01890 |
| B23L2 | Chr2_3270616 | C->T | exonic:synonymous | AT2G07675 |
| B23L2 | Chr2_8221770 | deletion(2): TC | UTR5 | AT2G18960 |
| B23L2 | Chr3_2609728 | deletion(1): C | intronic | AT3G08590 |
| B23L2 | Chr3_5568481 | C->A | UTR3 | AT3G16400 |
| B23L2 | Chr3_12426326 | deletion(1): A | transposable element | AT3TE51350 |
| B23L2 | Chr3_12806614 | C->T | intergenic |  |
| B23L2 | Chr3_14258703 | A->G | transposable element | AT3TE58605 |
| B23L2 | Chr3_15732338 | C->T | intergenic |  |
| B23L2 | Chr3_20062358 | T->A | exonic:nonsynonymous | AT3G54190 |
| B23L2 | Chr4_4418072 | C->T | intergenic |  |
| B23L2 | Chr4_5896901 | C->T | intergenic |  |
| B23L2 | Chr4_6081717 | C->T | transposable element | AT4TE25635 |
| B23L2 | Chr4_8669183 | deletion(1): A | UTR5 | AT4G15210 |
| B23L2 | Chr4_8844639 | G->C | intronic | AT4G15470 |
| B23L2 | Chr4_9870037 | deletion(3): TGT | exonic:nonframeshift | AT4G17750 |
| B23L2 | Chr4_16895578 | insertion(4): TATA | upstream;downstream |  |
| B23L2 | Chr4_17051455 | T->A | upstream;downstream |  |
| B23L2 | Chr4_17394158 | T->A | upstream |  |
| B23L2 | Chr4_17980533 | C->T | exonic:nonsynonymous | AT4G38405 |
| B23L2 | Chr5_7356477 | deletion(1): T | intronic | AT5G22200 |
| B23L2 | Chr5_9872614 | C->T | upstream |  |
| B23L2 | Chr5_10692053 | C->T | transposable element | AT5TE38935 |
| B23L2 | Chr5_12499229 | A->T | pseudogene | AT5G33251 |
| B23L2 | Chr5_12646742 | C->T | transposable element | AT5TE44770 |
| B23L2 | Chr5_13976747 | deletion(1): T | transposable element | AT5TE49800 |
| B23L2 | Chr5_14563554 | T->G | upstream;downstream |  |
| B23L2 | Chr5_23370350 | insertion(2): TT | intergenic |  |
| B23L3 | Chr1_12376491 | A->G | downstream |  |
| B23L3 | Chr1_12651414 | C->T | intronic | AT1G34550 |
| B23L3 | Chr1_13549753 | G->T | intronic | AT1G36180 |
| B23L3 | Chr1_14687243 | C->T | intergenic |  |
| B23L3 | Chr1_16389418 | G->A | transposable element gene | AT1G43502 |
| B23L3 | Chr1_17296298 | deletion(1): A | intergenic |  |
| B23L3 | Chr1_21055169 | deletion(1): T | intergenic |  |
| B23L3 | Chr1_26435332 | G->A | upstream |  |
| B23L3 | Chr2_275331 | A->G | upstream |  |
| B23L3 | Chr2_396945 | T->G | UTR3 | AT2G01890 |
| B23L3 | Chr3_2609728 | deletion(1): C | intronic | AT3G08590 |
| B23L3 | Chr3_5568481 | C->A | UTR3 | AT3G16400 |
| B23L3 | Chr3_12426326 | deletion(1): A | transposable element | AT3TE51350 |
| B23L3 | Chr3_12806614 | C->T | intergenic |  |
| B23L3 | Chr3_14258703 | A->G | transposable element | AT3TE58605 |
| B23L3 | Chr3_15732338 | C->T | intergenic |  |
| B23L3 | Chr3_20062358 | T->A | exonic:nonsynonymous | AT3G54190 |
| B23L3 | Chr4_4418072 | C->T | intergenic |  |
| B23L3 | Chr4_5896901 | C->T | intergenic |  |
| B23L3 | Chr4_6081717 | C->T | transposable element | AT4TE25635 |
| B23L3 | Chr4_8844639 | G->C | intronic | AT4G15470 |
| B23L3 | Chr4_9870037 | deletion(3): TGT | exonic:nonframeshift | AT4G17750 |
| B23L3 | Chr4_17051455 | T->A | upstream;downstream |  |
| B23L3 | Chr5_7356477 | deletion(1): T | intronic | AT5G22200 |
| B23L3 | Chr5_9872614 | C->T | upstream |  |
| B23L3 | Chr5_10692053 | C->T | transposable element | AT5TE38935 |
| B23L3 | Chr5_12499229 | A->T | pseudogene | AT5G33251 |
| B23L3 | Chr5_12646742 | C->T | transposable element | AT5TE44770 |
| B23L3 | Chr5_13976747 | deletion(1): T | transposable element | AT5TE49800 |
| B23L3 | Chr5_14563554 | T->G | upstream;downstream |  |
| B23L3 | Chr5_23370350 | insertion(2): TT | intergenic |  |
| B23L4 | Chr1_12376491 | A->G | downstream |  |
| B23L4 | Chr1_12651414 | C->T | intronic | AT1G34550 |
| B23L4 | Chr1_12736204 | insertion(1): A | UTR3 | AT1G34750 |
| B23L4 | Chr1_13549753 | G->T | intronic | AT1G36180 |
| B23L4 | Chr1_14687243 | C->T | intergenic |  |
| B23L4 | Chr1_16389418 | G->A | transposable element gene | AT1G43502 |
| B23L4 | Chr1_17296298 | deletion(1): A | intergenic |  |
| B23L4 | Chr1_21055169 | deletion(1): T | intergenic |  |
| B23L4 | Chr1_23153513 | A->G | exonic:nonsynonymous | AT1G62540 |
| B23L4 | Chr1_26435332 | G->A | upstream |  |
| B23L4 | Chr2_396945 | T->G | UTR3 | AT2G01890 |
| B23L4 | Chr3_2609728 | deletion(1): C | intronic | AT3G08590 |
| B23L4 | Chr3_5568481 | C->A | UTR3 | AT3G16400 |
| B23L4 | Chr3_12426326 | deletion(1): A | transposable element | AT3TE51350 |
| B23L4 | Chr3_12806614 | C->T | intergenic |  |
| B23L4 | Chr3_14258703 | A->G | transposable element | AT3TE58605 |
| B23L4 | Chr3_15732338 | C->T | intergenic |  |
| B23L4 | Chr3_20062358 | T->A | exonic:nonsynonymous | AT3G54190 |
| B23L4 | Chr3_22931096 | deletion(1): T | upstream |  |
| B23L4 | Chr4_4418072 | C->T | intergenic |  |
| B23L4 | Chr4_5896901 | C->T | intergenic |  |
| B23L4 | Chr4_6081717 | C->T | transposable element | AT4TE25635 |
| B23L4 | Chr4_8669183 | deletion(1): A | UTR5 | AT4G15210 |
| B23L4 | Chr4_8844639 | G->C | intronic | AT4G15470 |
| B23L4 | Chr4_9870037 | deletion(3): TGT | exonic:nonframeshift | AT4G17750 |
| B23L4 | Chr4_17051455 | T->A | upstream;downstream |  |
| B23L4 | Chr4_18434835 | G->A | downstream |  |
| B23L4 | Chr5_7356477 | deletion(1): T | intronic | AT5G22200 |
| B23L4 | Chr5_9872614 | C->T | upstream |  |
| B23L4 | Chr5_10692053 | C->T | transposable element | AT5TE38935 |
| B23L4 | Chr5_12499229 | A->T | pseudogene | AT5G33251 |
| B23L4 | Chr5_12646742 | C->T | transposable element | AT5TE44770 |
| B23L4 | Chr5_13976747 | deletion(1): T | transposable element | AT5TE49800 |
| B23L4 | Chr5_14563554 | T->G | upstream;downstream |  |
| B23L4 | Chr5_23370350 | insertion(2): TT | intergenic |  |
| B23L5 | Chr1_3776138 | A->C | intergenic |  |
| B23L5 | Chr1_12376491 | A->G | downstream |  |
| B23L5 | Chr1_12651414 | C->T | intronic | AT1G34550 |
| B23L5 | Chr1_12736204 | insertion(1): A | UTR3 | AT1G34750 |
| B23L5 | Chr1_13549753 | G->T | intronic | AT1G36180 |
| B23L5 | Chr1_14687243 | C->T | intergenic |  |
| B23L5 | Chr1_16389418 | G->A | transposable element gene | AT1G43502 |
| B23L5 | Chr1_17296298 | deletion(1): A | intergenic |  |
| B23L5 | Chr1_21055169 | deletion(1): T | intergenic |  |
| B23L5 | Chr1_26435332 | G->A | upstream |  |
| B23L5 | Chr2_396945 | T->G | UTR3 | AT2G01890 |
| B23L5 | Chr2_3139199 | G->A | intergenic |  |
| B23L5 | Chr2_6736816 | C->T | downstream |  |
| B23L5 | Chr2_10090761 | G->A | transposable element | AT2TE43165 |
| B23L5 | Chr2_12410908 | T->A | intergenic |  |
| B23L5 | Chr3_2609728 | deletion(1): C | intronic | AT3G08590 |
| B23L5 | Chr3_5568481 | C->A | UTR3 | AT3G16400 |
| B23L5 | Chr3_7250707 | T->C | intronic | AT3G20740 |
| B23L5 | Chr3_12426326 | deletion(1): A | transposable element | AT3TE51350 |
| B23L5 | Chr3_12806614 | C->T | intergenic |  |
| B23L5 | Chr3_14258703 | A->G | transposable element | AT3TE58605 |
| B23L5 | Chr3_15732338 | C->T | intergenic |  |
| B23L5 | Chr3_16848040 | G->A | exonic:stopgain | AT3G45840 |
| B23L5 | Chr3_20062358 | T->A | exonic:nonsynonymous | AT3G54190 |
| B23L5 | Chr3_22521170 | insertion(17): GGTGATTTTGCAGTTTT | upstream;downstream |  |
| B23L5 | Chr4_4418072 | C->T | intergenic |  |
| B23L5 | Chr4_5896901 | C->T | intergenic |  |
| B23L5 | Chr4_6081717 | C->T | transposable element | AT4TE25635 |
| B23L5 | Chr4_8669183 | deletion(1): A | UTR5 | AT4G15210 |
| B23L5 | Chr4_8844639 | G->C | intronic | AT4G15470 |
| B23L5 | Chr4_9870037 | deletion(3): TGT | exonic:nonframeshift deletion | AT4G17750 |
| B23L5 | Chr4_16895578 | insertion(4): TATA | upstream;downstream |  |
| B23L5 | Chr4_17051455 | T->A | upstream;downstream |  |
| B23L5 | Chr4_17737646 | deletion(1): T | intergenic |  |
| B23L5 | Chr5_7356477 | deletion(1): T | intronic | AT5G22200 |
| B23L5 | Chr5_9872614 | C->T | upstream |  |
| B23L5 | Chr5_10692053 | C->T | transposable element | AT5TE38935 |
| B23L5 | Chr5_11681536 | T->A | intergenic |  |
| B23L5 | Chr5_11972709 | C->T | intergenic |  |
| B23L5 | Chr5_12499229 | A->T | pseudogene | AT5G33251 |
| B23L5 | Chr5_12646742 | C->T | transposable element | AT5TE44770 |
| B23L5 | Chr5_13726315 | C->G | upstream |  |
| B23L5 | Chr5_13976747 | deletion(1): T | transposable element | AT5TE49800 |
| B23L5 | Chr5_14563554 | T->G | upstream;downstream |  |
| B23L5 | Chr5_23370350 | insertion(2): TT | intergenic |  |
| C20L1 | Chr1_14287354 | C->T | intergenic |  |
| C20L1 | Chr1_17744393 | T->A | exonic:nonsynonymous | AT1G48090 |
| C20L1 | Chr1_23674006 | G->A | intronic | AT1G63810 |
| C20L1 | Chr1_27474386 | G->T | ncRNA:antisense_lncRNA |  |
| C20L1 | Chr2_6435025 | G->A | intergenic |  |
| C20L1 | Chr2_9408483 | C->T | exonic:nonsynonymous | AT2G22125 |
| C20L1 | Chr2_9853582 | insertion(2): TA | upstream |  |
| C20L1 | Chr3_391261 | deletion(1): T | intronic | AT3G02150 |
| C20L1 | Chr3_12777098 | C->T | transposable element | AT3TE52540 |
| C20L1 | Chr3_14316857 | G->A | intergenic |  |
| C20L1 | Chr3_17999630 | deletion(1): T | intergenic |  |
| C20L1 | Chr3_20215397 | C->T | intronic | AT3G54610 |
| C20L1 | Chr4_2037989 | C->T | downstream |  |
| C20L1 | Chr4_5357833 | deletion(1): A | intergenic |  |
| C20L1 | Chr4_5592845 | deletion(40): TGATTTTGTATGGAACTCATAGTGAGTTTAATGTGATCAT | ncRNA:exonic |  |
| C20L1 | Chr4_6351404 | C->T | exonic:synonymous | AT4G10190 |
| C20L1 | Chr5_3815832 | insertion(2): TA | upstream;downstream |  |
| C20L1 | Chr5_6440878 | G->A | intergenic |  |
| C20L1 | Chr5_11212448 | T->C | intergenic |  |
| C20L1 | Chr5_24945114 | T->C | exonic:nonsynonymous | AT5G62110 |
| C20L1 | Chr5_25824649 | deletion(1): T | transposable element | AT5TE92940 |
| C20L2 | Chr1_12248544 | deletion(1): A | intergenic |  |
| C20L2 | Chr1_13660657 | C->T | intergenic |  |
| C20L2 | Chr1_14287354 | C->T | intergenic |  |
| C20L2 | Chr1_17744393 | T->A | exonic:nonsynonymous | AT1G48090 |
| C20L2 | Chr1_23674006 | G->A | intronic | AT1G63810 |
| C20L2 | Chr2_5521440 | insertion(1): C | intergenic |  |
| C20L2 | Chr2_6435025 | G->A | intergenic |  |
| C20L2 | Chr2_9408483 | C->T | exonic:nonsynonymous | AT2G22125 |
| C20L2 | Chr2_9853582 | insertion(2): TA | upstream |  |
| C20L2 | Chr3_1787812 | C->T | exonic:synonymous | AT3G05970 |
| C20L2 | Chr3_12777098 | C->T | transposable element | AT3TE52540 |
| C20L2 | Chr3_13992877 | C->T | intergenic |  |
| C20L2 | Chr3_14316857 | G->A | intergenic |  |
| C20L2 | Chr3_17999630 | deletion(1): T | intergenic |  |
| C20L2 | Chr3_20215397 | C->T | intronic | AT3G54610 |
| C20L2 | Chr4_5357833 | deletion(1): A | intergenic |  |
| C20L2 | Chr4_5592845 | deletion(40): TGATTTTGTATGGAACTCATAGTGAGTTTAATGTGATCAT | ncRNA:exonic |  |
| C20L2 | Chr4_6351404 | C->T | exonic:synonymous | AT4G10190 |
| C20L2 | Chr5_3815832 | insertion(2): TA | upstream;downstream |  |
| C20L2 | Chr5_6440878 | G->A | intergenic |  |
| C20L2 | Chr5_11212448 | T->C | intergenic |  |
| C20L2 | Chr5_11795972 | T->A | intergenic |  |
| C20L2 | Chr5_11795973 | G->T | intergenic |  |
| C20L2 | Chr5_15790538 | deletion(1): A | upstream;downstream |  |
| C20L2 | Chr5_17168852 | T->A | exonic:synonymous | AT5G42810 |
| C20L2 | Chr5_20675979 | G->A | exonic:nonsynonymous | AT5G50810 |
| C20L2 | Chr5_23038706 | G->A | exonic:synonymous | AT5G56960 |
| C20L2 | Chr5_24945114 | T->C | exonic:nonsynonymous | AT5G62110 |
| C20L2 | Chr5_25824649 | deletion(1): T | transposable element | AT5TE92940 |
| C20L2 | Chr5_26755913 | T->C | intronic | AT5G67030 |
| C20L3 | Chr1_14287354 | C->T | intergenic |  |
| C20L3 | Chr1_17744393 | T->A | exonic:nonsynonymous | AT1G48090 |
| C20L3 | Chr1_23674006 | G->A | intronic | AT1G63810 |
| C20L3 | Chr2_379447 | C->T | intergenic |  |
| C20L3 | Chr2_6435025 | G->A | intergenic |  |
| C20L3 | Chr2_9408483 | C->T | exonic:nonsynonymous | AT2G22125 |
| C20L3 | Chr3_12777098 | C->T | transposable element | AT3TE52540 |
| C20L3 | Chr3_14316857 | G->A | intergenic |  |
| C20L3 | Chr3_17999630 | deletion(1): T | intergenic |  |
| C20L3 | Chr3_20215397 | C->T | intronic | AT3G54610 |
| C20L3 | Chr4_1507245 | C->T | upstream |  |
| C20L3 | Chr4_5357833 | deletion(1): A | intergenic |  |
| C20L3 | Chr4_5592845 | deletion(40): TGATTTTGTATGGAACTCATAGTGAGTTTAATGTGATCAT | ncRNA:exonic |  |
| C20L3 | Chr4_6351404 | C->T | exonic:synonymous | AT4G10190 |
| C20L3 | Chr5_3815832 | insertion(2): TA | upstream;downstream |  |
| C20L3 | Chr5_6440878 | G->A | intergenic |  |
| C20L3 | Chr5_9952143 | C->G | intergenic |  |
| C20L3 | Chr5_11212448 | T->C | intergenic |  |
| C20L3 | Chr5_24945114 | T->C | exonic:nonsynonymous | AT5G62110 |
| C20L3 | Chr5_25824649 | deletion(1): T | transposablev element | AT5TE92940 |
| C20L4 | Chr1_648035 | T->C | intronic | AT1G02890 |
| C20L4 | Chr1_14287354 | C->T | intergenic |  |
| C20L4 | Chr1_14461705 | T->A | intergenic |  |
| C20L4 | Chr1_17744393 | T->A | exonic:nonsynonymous | AT1G48090 |
| C20L4 | Chr1_22878972 | insertion(2): TG | intergenic |  |
| C20L4 | Chr1_23674006 | G->A | intronic | AT1G63810 |
| C20L4 | Chr2_6435025 | G->A | intergenic |  |
| C20L4 | Chr2_9408483 | C->T | exonic:nonsynonymous | AT2G22125 |
| C20L4 | Chr2_9853582 | insertion(2): TA | upstream |  |
| C20L4 | Chr2_11050395 | G->A | exonic:nonsynonymous | AT2G25910 |
| C20L4 | Chr2_12720611 | A->G | downstream |  |
| C20L4 | Chr3_12777098 | C->T | transposable element | AT3TE52540 |
| C20L4 | Chr3_13410839 | G->A | intergenic |  |
| C20L4 | Chr3_14316857 | G->A | intergenic |  |
| C20L4 | Chr3_15135761 | C->T | transposable element | AT3TE61600 |
| C20L4 | Chr3_17999630 | deletion(1): T | intergenic |  |
| C20L4 | Chr3_20215397 | C->T | intronic | AT3G54610 |
| C20L4 | Chr4_2037989 | C->T | downstream |  |
| C20L4 | Chr4_5357833 | deletion(1): A | intergenic |  |
| C20L4 | Chr4_5592845 | deletion(40): TGATTTTGTATGGAACTCATAGTGAGTTTAATGTGATCAT | ncRNA:exonic |  |
| C20L4 | Chr4_6351404 | C->T | exonic:synonymous | AT4G10190 |
| C20L4 | Chr5_3815832 | insertion(2): TA | upstream;downstream |  |
| C20L4 | Chr5_6440878 | G->A | intergenic |  |
| C20L4 | Chr5_11212448 | T->C | intergenic |  |
| C20L4 | Chr5_24945114 | T->C | exonic:nonsynonymous | AT5G62110 |
| C20L4 | Chr5_26755913 | T->C | intronic | AT5G67030 |
| C20L5 | Chr1_9543212 | T->G | intronic | AT1G27470 |
| C20L5 | Chr1_14287354 | C->T | intergenic |  |
| C20L5 | Chr1_17744393 | T->A | exonic:nonsynonymous | AT1G48090 |
| C20L5 | Chr1_23674006 | G->A | intronic | AT1G63810 |
| C20L5 | Chr2_2239439 | G->A | intergenic |  |
| C20L5 | Chr2_4415808 | C->T | intergenic |  |
| C20L5 | Chr2_5521440 | insertion(1): C | intergenic |  |
| C20L5 | Chr2_6435025 | G->A | intergenic |  |
| C20L5 | Chr2_8469523 | C->T | upstream;downstream |  |
| C20L5 | Chr2_9408483 | C->T | exonic:nonsynonymous | AT2G22125 |
| C20L5 | Chr2_9853582 | insertion(2): TA | upstream |  |
| C20L5 | Chr3_12777098 | C->T | transposable element | AT3TE52540 |
| C20L5 | Chr3_14316857 | G->A | intergenic |  |
| C20L5 | Chr3_17999630 | deletion(1): T | intergenic |  |
| C20L5 | Chr3_20215397 | C->T | intronic | AT3G54610 |
| C20L5 | Chr4_5068668 | G->A | transposable element | AT4TE21090 |
| C20L5 | Chr4_5357833 | deletion(1): A | intergenic |  |
| C20L5 | Chr4_5592845 | deletion(40): TGATTTTGTATGGAACTCATAGTGAGTTTAATGTGATCAT | ncRNA:exonic |  |
| C20L5 | Chr4_6059381 | deletion(1): T | upstream |  |
| C20L5 | Chr4_6351404 | C->T | exonic:synonymous | AT4G10190 |
| C20L5 | Chr4_7807195 | insertion(3): AGA | intronic | AT4G13430 |
| C20L5 | Chr5_6440878 | G->A | intergenic |  |
| C20L5 | Chr5_3815832 | insertion(2): TA | upstream;downstream |  |
| C20L5 | Chr5_11212448 | T->C | intergenic |  |
| C20L5 | Chr5_24945114 | T->C | exonic:nonsynonymous | AT5G62110 |
| C20L5 | Chr5_26755913 | T->C | intronic | AT5G67030 |

**Table S4.** Simulated reference mutations recovered from six randomly selected MA lines.

| Sample | Accessible reference sites (proportion of TAIR10 genome) | Recovered SNVs | Expected SNVs | Recovered SNVs/Expected SNVs |
| --- | --- | --- | --- | --- |
| A17L2 | 114417683 (95.61%) | 100 | 100 | 1.00 |
| B23L5 | 114660173 (95.82%) | 99 | 100 | 0.99 |
| C20L2 | 113743864 (95.05) | 95 | 100 | 0.95 |
| D11L4 | 117406104 (98.11%) | 98 | 100 | 0.98 |
| E11L4 | 117251673 (97.98%) | 99 | 100 | 0.99 |
| F11L4 | 117376048 (98.08%) | 97 | 100 | 0.97 |

**Table S5.** Simulated sequence read mutations recovered from six randomly selected MA lines.

| Sample | Simulated homozygous SNMs | Called heterozygous SNVs | Called  homozygous SNVs | Homozygous SNVs/Simulated |
| --- | --- | --- | --- | --- |
| A17L2 | 100 | 1 | 77 | 0.77 |
| B23L5 | 100 | 4 | 83 | 0.83 |
| C20L2 | 100 | 4 | 71 | 0.71 |
| D11L4 | 100 | 1 | 89 | 0.89 |
| E11L4 | 100 | 0 | 91 | 0.91 |
| F11L4 | 100 | 0 | 87 | 0.87 |

**Table S6.** Validation of mutations detected in MA lines through conventional Sanger sequencing.

| Sample | Chr_Position | Mutation* | Forward primer (5'-3') | Reverse primer (5'-3') | Sequencing Results |
| --- | --- | --- | --- | --- | --- |
| D10L1 | Chr1_28180441 | A->G | ATAATTCTCCAAAACATATCGTG | AGAACCGTCCTGACCATCC | Confirmed |
| D10L1 | Chr1_30045923 | G->A | ACTGTGGAAACACGGAATG | TCTGGGATGGTTAATGACTT | Confirmed |
| D10L1 | Chr2_9481938 | Del (6 bp) | ATTGGAAGGAAGTCAAGGGTT | GACAGCACCGGACCACTCA | PCR failure |
| D10L1 | Chr3_3299184 | Del (1 bp) | TCCGCAATCGTTTCCCCCTT | TGGCCGATCAGCTCGAGAGG | Confirmed |
| D10L1 | Chr5_21796347 | G->A | AAACCAATAGTGAAAAGGGACA | ACGCTTCGTAAAAGAAAGAACA | Confirmed |
| D10L2 | Chr1_12741653 | C->T | TGGTGTAGTGATCCCATGCTC | TGATTAGTGTTTTGCCTCGGT | Confirmed |
| D11L2 | Chr1_8500098 | Ins (1 bp) | ACGATTATCCAAAACCATTAGG | CAATTTGGTGAAGGAAGCTCAA | Confirmed |
| D11L2 | Chr1_10021083 | T->G | AAGTTCGTCAAATCGGTGATACAT | GGTTGATGAGAGAGTTTGTTCCTA | Confirmed |
| D10L2 | Chr1_20755080 | G->A | ACGGAAAGTGATGTTGAGAA | AACCAAGCAGCTAAGGATTA | Confirmed |
| D11L2 | Chr3_6651225 | Ins (2 bp) | CATAAGTCCACCTTGTCCGCAT | GTCTTTTGTCTGCTCGTTTGCT | Confirmed |
| D11L2 | Chr3_12090975 | G->A | TGGTTGTAGACGTGGAGAC | TCTATGCTACTTACGCTTT | Confirmed |
| D10L2 | Chr3_13795890 | A->G | GAGGTTACCCATCTTCGTACT | CGTGTTGCATCCCTCATTT | Confirmed |
| D11L2 | Chr3_20040388 | A->G | TGAGACAGGTAGTTATCCCGAC | GAACAATGGAGGTATCAGAAGC | Confirmed |
| D10L2 | Chr5_8812541 | G->A | AGGCTGTTTTGAATCGCTTA | AACGTCATTGTAACAAATTGATG | Confirmed |
| D10L2 | Chr5_22379192 | T->A | ATTCGCTTTTAATAGGTTTTGAG | TGCTTTTACATCCTTCCGT | Confirmed |
| D10L3 | Chr1_5277948 | A->G | GGCTTTCTTTCCTTTCTGGTA | ATTAGGGATTAGACTTGTGCC | Confirmed |
| D10L3 | Chr1_15903567 | T->C | AATAAGGGAATACATCGGTTAT | ATAGAAGAATGAACATGCGAAT | Confirmed |
| D10L3 | Chr2_17323097 | Ins (1bp) | CAATACAGCAAATGATACCAGC | TCAGCAACAGCACTAATCTTAA | Confirmed |
| D10L3 | Chr3_9444815 | G->T | AGCCATACTCGAAAGGGAATT | CGGTACAAGTGAACGGTGAA | Confirmed |
| D11L3 | Chr4_388431 | Ins (3 bp) | TTAGGGTTTATGTTACCTCTTTCCG | GTTTGAAGCGGGTCAGATGG | Confirmed |
| D10L3 | Chr4_8020162 | A->G | TATAACCGTGAATAAGGTTGCT | AAGTTTTCAATCTGCTTCCTA | Confirmed |
| D10L3 | Chr5_8088478 | Del (1 bp) | TCCGTTATCACCATACCTCTTT | GCAGTATTTCTTTGCCACCTTC | Confirmed |
| D10L3 | Chr5_13481434 | G->C | CAGAAACCTAACCCAATGACCA | CCAAATCAAGTAAATGGAGTGC | Confirmed |
| D10L3 | Chr5_19141355 | G->A | AAGGTTTTCACTTCTGCTTCTC | TGTTCACCACTATACATCCCAC | Confirmed |
| D10L4 | Chr1_13264193 | C->T | GCCGACCTTGGAAACATAA | AGCAAACCCATCCTGCTCT | Confirmed |
| D10L4 | Chr2_2740878 | C->T | ATGTAGTTCTTCGGGATCTTT | AAGCCATCTATGGACTGAAAC | Confirmed |
| D11L4 | Chr3_2550672 | A->T | GGACTTGAAAATCCAGACCGTA | AAAGGAGCTTATGGTCGAGTTT | Confirmed |
| D10L4 | Chr3_4835343 | A->T | ATCATCGTCACTTTTGGATAT | GTGCAAGATTTTGTTTACATTAC | Confirmed |
| D11L4 | Chr3_13858270 | T->C | CTAGGATGAGTGAGCTCCAGGA | AGGTCACCCATCCTCGTACT | Confirmed |
| D10L4 | Chr5_3805033 | T->C | AGAATATGAGGAAGATGATGGCTAA | AGGACTGTGCTGTGGGTTTG | Confirmed |
| D10L4 | Chr5_12023299 | A->C | ATCTGGTGCTTTGGCTCTG | AAATTCCAGGATGGCAGTA | Confirmed |
| D10L4 | Chr5_16014724 | G->C | AAGAAGACACTTTAAAACCCT | GACCAAATTATCTTCATCTAAT | Confirmed |
| D10L5 | Chr1_7080936 | C->T | GTCAAATAATGATAACCAGGAC | GCTACTTCCGTATCTCGCATA | Confirmed |
| D10L5 | Chr1_12024468 | T->C | TCAAAAGGATCATTTCAGTG | AACAAGGGATAGCCAGAGTT | Confirmed |
| D10L5 | Chr1_15563567 | G->A | GTGGACGGTCGTAGTAAATG | TTCATCGTCTTTGTCCCTTA | Confirmed |
| D10L5 | Chr2_5891163 | G->A | TCTGGGATTGTTGGCGACT | GGCAGCGGTTGTTTCTTC | Confirmed |
| D11L5 | Chr2_13702918 | Del (1 bp) | ACTTGTGTGCTACATAGGTCCG | CGAAGCCTCCGATAACGATT | Confirmed |
| D11L5 | Chr2_19697643 | G->A | GCTCAAGTGGTTTGCTTTGGT | CTCAGACCCCAAACCCACAG | PCR failure |
| D10L5 | Chr5_15272931 | C->T | ATTTTAGGGCACCTTTACATA | GGAGATTATTTGTATTCGCTTTT | Confirmed |
| E10L1 | Chr1_4299919 | T->A | TGTTTCTACTTTCTTGTATCGTTGA | AGACTCGAAACAAATTGATCATG | Confirmed |
| E10L1 | Chr1_17440330 | C->T | TACCGTTCAAGACCCTTTTCG | AGCATTAGCCATCAGAGTTCG | Confirmed |
| E10L1 | Chr1_21858117 | C->T | ACAAACCAAAGGTCTCACATC | GCCTTTCGCTTAGAAATAGGG | Confirmed |
| E10L1 | Chr2_509829 | A->G | CAATGCTTAGTCTTTGTTCTTGT | ATCCTTTGAGGATATGGTAGG | Confirmed |
| E10L1 | Chr2_3232467 | C->G | GGCTCGTCGTATCCATTTCAT | GCGGATTTGTCGGGAGTT | Confirmed |
| E10L1 | Chr2_4041454 | C->T | AACCACAACGTCAAGCTCC | CCACAACAAGCCATTCTACAT | Confirmed |
| E10L1 | Chr2_12003173 | Del (1 bp) | AGAGTTGTCATATTTGTTGGAGT | CTTTTGAGCAACATTGGATTT | Confirmed |
| E10L1 | Chr3_4368321 | C->T | ATCACGACCAACATTCTACCA | CAATGCCTCAACCGCTTCT | Confirmed |
| E10L1 | Chr3_17912372 | Del (1 bp) | ACTCAATCTGTGTCAACGGAAT | TGCGGAAACTTATCAACAAACT | Confirmed |
| E10L1 | Chr4_112973 | G->A | TCATTACAACCATCGACCAA | AGAAGTGGAGCAAGGTGTCT | Confirmed |
| E10L1 | Chr4_4060978 | G->A | AAACACCCTTAATCCAACTG | TCTCCCAGAAGATGCTAA | Confirmed |
| E10L1 | Chr4_10783884 | A->G | TGTTACTGCGACAATCGA | TAACCCTAATCAAGCCAAC | Confirmed |
| E10L1 | Chr4_11368743 | C->T | AGTCAACTTAATGAAACCCTC | CGTTATCGCCGTATGCTC | Confirmed |
| E10L1 | Chr4_14450247 | C->T | TTGGAAGGCTAAATCGTGT | AGATCGGATCACAAGGAATAA | Confirmed |
| E10L1 | Chr4_17786593 | Del (1 bp) | TGCCTGAGACTAACCTGTCAAC | TTTCTCGGGTTCTTCTTCTTTA | Confirmed |
| E10L1 | Chr5_8721045 | C->A | ATCTTGTAAAGAACCGAGCAT | TGGAAGGTTTTCAAACGAA | Confirmed |
| E10L1 | Chr5_10735764 | G->A | TGAGACCCAGCGATGAAAG | CACCTACGTGCATCTTCGTCTC | Confirmed |
| E10L1 | Chr5_13526118 | Ins (1bp) | AAGAAGGCTCTAAAGATCGTTG | CCTCCATAGGATTACACATCAT | Confirmed |
| E10L1 | Chr5_15646457 | G->A | GGAATTTGGTTAAGCGTCAT | CTGCGATTCAAACTTGGTGT | Confirmed |
| E11L1 | Chr5_18040165 | Del (1 bp) | AAAAAGGAAACCCGATTGCTAC | GTTCGTCTGATGACACACTTGA | Confirmed |
| E10L2 | Chr1_1592311 | A->T | ATTGCTTCTTCACTCACCAT | ATCCAACCAACTCTGCTTAT | Confirmed |
| E11L2 | Chr1_2481570 | T->C | TTGAGTCTTGTGATGAATGGTC | TCCTGAGAGAAATACAAAGACG | Confirmed |
| E10L2 | Chr1_7312386 | T->C | AAACAACGTGTAGAGGCGATAA | AGTTGCCATTAGTTTGGTAAGC | Confirmed |
| E10L2 | Chr1_16140730 | Del (1 bp) | CCGACCTTTTCAGTGTCTTATA | GAACAAGTCACGAAGTAATCAG | Confirmed |
| E10L2 | Chr1_17863094 | G->A | GTAAGTGTTTGGTGTTTGTCG | TGGTCTAACGAGTTTTGGAGT | Confirmed |
| E10L2 | Chr1_18097747 | Del (4 bp) | TTTTGTAGCCTCGTTAAGTT | GTATAACTGTCTTTGCAGCATT | Confirmed |
| E10L2 | Chr3_9739441 | G->A | ATAATGCTGACCACTTCGTT | GCTATTACACGCTCTTCTAT | Confirmed |
| E11L2 | Chr3_13783756 | T->A | AGATCACGAAAAGGCCCGAA | CATGCGATCATACCAGCACT | Confirmed |
| E10L2 | Chr3_14249144 | C->G | CGTTTATTACCGAGCAAGGA | TAATGCGGCAATACGACCAG | Confirmed |
| E10L2 | Chr3_17451284 | G->C | TTGTGCTTTGTCTTCGTAAG | AACACTATCACCAATGTCGG | Confirmed |
| E11L2 | Chr4_3507964 | Ins (15 bp) | CTTCGCATCGAGTTGTCCATTC | TTGTAGAGTTGGTCGCCAATGA | PCR failure |
| E10L2 | Chr4_9872289 | C->A | TCAAGGTAGGCCGAGATT | TGCTCAAAGTTGTAGTCCC | Confirmed |
| E10L2 | Chr4_11591112 | Del (1 bp) | TTGATTCTAGTCATTTACACCT | TTGTTGTTTGTCCCTTGTATGG | Confirmed |
| E10L2 | Chr5_8320799 | G->T | CTCTATAAGATCAATGCAGGAA | GTGATGTAAATGCTTTACGAAG | Confirmed |
| E10L2 | Chr5_8320806 | T->C | CTCTATAAGATCAATGCAGGAA | GTGATGTAAATGCTTTACGAAG | Confirmed |
| E10L2 | Chr5_8320843 | A->C | CTCTATAAGATCAATGCAGGAA | GTGATGTAAATGCTTTACGAAG | Confirmed |
| E10L2 | Chr5_11310914 | Del (1 bp) | TTGTATTGAATGAACTAACGGA | TAGAGTCATATCGTGTTAACCG | Confirmed |
| E10L2 | Chr5_13744565 | C->T | ATGCCACTTCATTAGGTCTATG | GCATTCTAGGACCAGTGCAGAT | Confirmed |
| E10L2 | Chr5_13942107 | C->T | CCTCCCAGACATTGTTCATCTT | CAATAACTCAGCCATCAAATCG | Confirmed |
| E10L3 | Chr1_5841059 | A->T | CCTTCAGCAACACCACCAT | GCAGATTTGGGAATTTAGG | Confirmed |
| E11L3 | Chr1_11957865 | Del (1 bp) | AAGAAACCTGAGCCGCTACAAT | TTCGCTAACTCATCCGCTAGAC | Confirmed |
| E10L3 | Chr1_13301299 | G->A | TATAAATCCTATAAAACGTCCAG | CAGTGGGACAACTATGGAGAT | Confirmed |
| E10L3 | Chr1_24553233 | T->A | CGGTTATCGTTAAACCAGTCG | CATTCACGGTGTCCATAGTTC | Confirmed |
| E10L3 | Chr2_1761184 | Del (3 bp) | ATTTTGTGCCACCGTCTTATCA | TATTTCGTCTGTCTCTTCCTCG | Confirmed |
| E10L3 | Chr2_4995013 | T->A | AAAGTGATGGAGGTGAAGGAA | GATACGCGAGGTAGATTGCTG | Confirmed |
| E10L3 | Chr2_5037964 | T->A | GATGCGAATAGTGTACCTTGA | TAACGTCCTGTCAAACCCGTC | Confirmed |
| E10L3 | Chr2_5414127 | C->T | TTACTATCACAGGCCAAATCG | AGTTAGAGGCTACCGCAAAGG | Confirmed |
| E10L3 | Chr2_5536209 | A->G | CATTGGAGGTGGTCTTAGATG | GTAAGACATGGGGAAAAGGTA | Confirmed |
| E10L3 | Chr2_8060293 | Ins (1 bp) | GGGGGGAGTTACAAGGGAA | TGTGACAAAAACCCACCGAT | Confirmed |
| E10L3 | Chr2_16333363 | T->C | TAAAGGCATTGGGTTGGAAGA | TAAGTTACATATCTGAGGTGGTG | Confirmed |
| E10L3 | Chr2_16405434 | G->A | CCTGCTCTATGTCGTTTCCTA | AGTCTTCAAAGGGAGTATGTG | Confirmed |
| E10L3 | Chr3_16830078 | T->A | GGCTTTTCGGGTTTCATTAGTT | TGAATCACTCGTTCGTTCTTTT | Confirmed |
| E10L3 | Chr3_18945978 | T->A | CTCAGCTCTTCCGGGTTTCT | GACCGAGTCAAAGGTTTCATA | Confirmed |
| E10L3 | Chr3_19139288 | Del (1 bp) | ACAACTTCAAGAGTCCCTCCA | GTACCAGCGAGGCCAGAC | Confirmed |
| E10L3 | Chr3_20098935 | G->A | TGGGTCCATTGTGCTCATC | TTCCAGCACTTTCTTTGTTCC | Confirmed |
| E10L3 | Chr3_21509911 | Del (2 bp) | GCATAGATCCGATTTGTGACCC | GGGGACCACCTTTGTGTGATTT | Confirmed |
| E10L3 | Chr4_5555374 | A->C | AACTGGTGGTGATAGACGG | CTGCGATTTGATTGAACGA | Confirmed |
| E11L3 | Chr4_18096196 | Del (2 bp) | GGTCATCGGAGAGAGATGCATT | CGTAGATGGGTTTTTAGGCCCT | PCR failure |
| E10L3 | Chr5_1072079 | C->T | GCACCACTTTGTTGAAATCCC | AAACCGATGATGGGTCTTTAC | Confirmed |
| E10L3 | Chr5_16154747 | C->A | ATCTCAAATCCACCATTGTTTC | GGTTAGCATACGGATCACTTC | Confirmed |
| E10L3 | Chr5_19774483 | Del (1 bp) | AAGCTCAGCTAAGCGGTAT | GTGCCTCTTTCCAGTGTTAT | Confirmed |
| E10L3 | Chr5_26949851 | C->T | TTCACTCATACATAGAGTCCCACA | GCCTTTGTGGATTATTAGTTGGT | Confirmed |
| E10L4 | Chr1_752141 | T->C | AAGGTCCACATCAATTTAACTAACC | TTGCTTTCTTGCCACGTCAT | Confirmed |
| E10L4 | Chr1_11509302 | G->T | GGAACAGAACACGGCAACAT | CGGGAGTTAAGAAGGCGATT | Confirmed |
| E10L4 | Chr1_16038306 | C->T | ATGCCTTTATCGAGTTCTTTGG | TTGTCCCACTGCATAGAATACC | Confirmed |
| E10L4 | Chr1_18941043 | Del (1 bp) | ACATTTCTTACCTCATCTGGACG | GCACCAACAAGGTAAAATCACA | Confirmed |
| E10L4 | Chr1_22224924 | G->A | ATCTATCCGCAGAAATTGGA | TGAAAAGTGAGGAAACAAGA | Confirmed |
| E10L4 | Chr1_24896437 | G->A | TACATGGTACAGAGCGCCTGAG | TAATGGTGGAGCGGGTGTAT | Confirmed |
| E10L4 | Chr1_26847185 | Del (1 bp) | TTTTACGAGCGTGAATATCTG | CAAGTCCAAGTTCCCAAGACA | Confirmed |
| E10L4 | Chr2_1655747 | Del (2 bp) | CTAATCGCACTGCTCTCATAGA | TGGTCCTTGTAGATCGACTTCG | Confirmed |
| E10L4 | Chr2_4351715 | Del (7 bp) | AGGGAAACAATTTTGAGGAG | GGGGTTAGTTGGTAGGACGG | Confirmed |
| E10L4 | Chr2_5198118 | A->T | AACGATGGCGTCTTTGTATT | GGAGGGTTTAGTTAAGGAAT | Confirmed |
| E10L4 | Chr2_8680343 | C->T | GACTTGTCTTTTCCAAACTACC | TTGGTGCATCATATTGTTCCTC | Confirmed |
| E10L4 | Chr3_10719893 | Del (1 bp) | ATGTCTATACGGGACTGGTGAG | CATCTATGGCTAATGAGGTAACTT | Confirmed |
| E10L4 | Chr3_12717669 | C->T | ATCCGTATGCTCAGATGTCAGG | CACAGTCCATTCGGTTAGGTGA | Confirmed |
| E10L4 | Chr3_13525199 | Del (1 bp) | AAGCATACATCCTATTATCGTCAC | GATTAGTCTCTTTGCCTTATCTTG | Confirmed |
| E10L4 | Chr3_13693107 | G->T | TATGATTCATTTACCGTGGGTC | TTATGGCATTTTCGTGGTCTTT | PCR failure |
| E10L4 | Chr4_963036 | C->T | GATAGATGGTCCAATGGTTTC | TTTCGCCATAGCATAACAAGAG | Confirmed |
| E10L4 | Chr4_3562787 | G->C | ATAATGATGGTGGGTTTCTAG | GTACCTACGTTTCGCCTATT | Confirmed |
| E11L4 | Chr5_8320803 | G->A | CTCTATAAGATCAATGCAGGAA | GTGATGTAAATGCTTTACGAAG | Confirmed |
| E10L4 | Chr5_12203220 | T->C | CGTGAAGTATGGCTATCAGAGG | GACATACATAATCTTTCACCCA | Confirmed |
| E10L4 | Chr5_12262232 | T->A | GATTTGTACTTAATGTGCTTCA | TCTTCGTTGGCAAAGGAACT | Confirmed |
| E11L5 | Chr1_1933382 | Del (1 bp) | AAAGGGGTTCAAGTTCTGGTTA | GTCATCTCAGCCGACGAACTAC | Confirmed |
| E10L5 | Chr1_13297903 | G->A | ATGAGTCTATTTCGTTGCCAATC | TCAAAAGTCAGGAACTAACCGT | Confirmed |
| E10L5 | Chr1_18132357 | G->C | TGTTCGTTTGTTAGTCGTTG | AGTAAGAGGTTGATTGGGAC | Confirmed |
| E10L5 | Chr1_21128436 | T->A | TATTCTTACTAAACGTCTTTTG | TTATTAAGTAACGTCCTGAC | Confirmed |
| E10L5 | Chr2_3051713 | T->G | GATTAAATAAACAAGCGTAC | TTTTCTTTCACAGGCAACTC | Confirmed |
| E10L5 | Chr2_13868472 | G->A | AAGACAACTGCCAGTGGATC | GACCACTCACTCCAGGGTTT | Confirmed |
| E10L5 | Chr3_234616 | Del (1 bp) | TTCTTTATGTACCCCTCCCCAA | GAGCCTATCATCTCCTCCACTG | Confirmed |
| E10L5 | Chr3_9785615 | G->A | TATAACCCGCATCAAACCTA | GATAGCGTCAGATGGGATTT | Confirmed |
| E10L5 | Chr3_12752365 | G->A | ATACGGATTCATGCCTCTGA | CCCACTATGCGCTTGTTTAT | Confirmed |
| E10L5 | Chr4_1507947 | Ins (2 bp) | TTCCCTCCCTTGTATTTCCTAA | ATGAAAGGAAAAAGAGAAATGG | Confirmed |
| E10L5 | Chr4_9377511 | A->G | TCTGAAACGAGGACAGAGTC | AGTAAGGTGGAACTGAAACAA | Confirmed |
| E10L5 | Chr4_12102010 | T->G | CTATTACTGTAGCCAAGGCACT | CGACTATAACTAGCGACTGG | Confirmed |
| E10L5 | Chr4_14602866 | Del (1 bp) | TTCCGAACACGAGTAAAGCC | ACCGTCGTAGGAGACTCATAACAT | Confirmed |
| E10L5 | Chr5_1383661 | Del (1 bp) | AAAAGGGTTCAACTCTAAGCGT | ATTTTCGTGGTCCCGTGTTCT | Confirmed |
| E10L5 | Chr5_7909462 | Del (2bp) | ATCTCTCTGCTCTTGAGTTCCG | TAAATCCGAAAGGAGGGGGC | Confirmed |
| E10L5 | Chr5_12089326 | Del (1 bp) | TCCATTGAACAACTAATATGTAGA | CGCAAAAACCTTAATTTAGAGAGA | Confirmed |
| E10L5 | Chr5_20729723 | A->G | TCGTGCTCCACCATTAGACAAAG | CGTCTGTGCTCCCCATAATCT | Confirmed |
| E10L5 | Chr5_21660951 | Del (1 bp) | AGTGCATTAATCAGAAATCATG | CAGAATAAGAATGAGGAATAGG | Confirmed |
| F11L1 | Chr1_6677325 | Ins (1 bp) | CCGACACTGGATTATCACAAGC | TCCGGCCAACCTTAACCCT | Confirmed |
| F10L1 | Chr1_9094442 | C->A | TATGAAAACCTCAGTAACCCTA | TCACGATTAACAAAAGCGAAAA | Confirmed |
| F10L1 | Chr1_12130437 | A->G | TGTATTATGTCAAACAAAGGAA | TCCATTTCTTCCCACTACTCTA | Confirmed |
| F10L1 | Chr2_3966820 | C->T | AATCCGACTCTTATGTTGGACC | TCCGCTAGATGTAACAAAGTGTTGAG | Confirmed |
| F10L1 | Chr3_16939107 | G->A | GAGCCTACTCCATACTTTCA | TAGCGACAACTTCGTCCTAT | Confirmed |
| F10L1 | Chr3_20175685 | A->G | AGAAAGGAGCAGTCAGGAGA | TCCTAAATGCAGCATACGAG | Confirmed |
| F10L1 | Chr4_3678327 | G->A | CGAGATAAAGATAAAGAGGGAT | AACTACGCTACTGGAGACTTG | Confirmed |
| F10L1 | Chr4_6635396 | C->T | TGGTCAGACTACGAAGGCTAT | TCGGAGTTGGTCGCCTTG | Confirmed |
| F10L1 | Chr5_5229726 | Ins (3 bp) | GTTCATTTCCGTTCACCGTAAG | AGTCGTTGTCTGAGCAAGCGTA | Confirmed |
| F10L1 | Chr5_5933484 | A->T | CAAACCAGTGTCGAGGAAGC | CCATATCGTGCATAAACTACGT | Confirmed |
| F10L1 | Chr5_14023478 | G->A | CCACGGGTAGTCTCGGAAC | CCCAGGTTTATCGGGACTTA | Confirmed |
| F10L1 | Chr5_24779066 | Del (2 bp) | GTTCGGAATTGGGTTTGAGT | CCTTTGCCTTGTATGCTTTT | Confirmed |
| F10L2 | Chr1_26011710 | Del (1 bp) | CGTAATAGGGTAATCGTGGACT | TGAAGTTATACGAGCATGGAAA | Confirmed |
| F10L2 | Chr1_27740161 | C->T | AGTCCATCAGGGTAAGGCAGTC | TTTAGGTTTATCGTCTTTTCGT | Confirmed |
| F11L2 | Chr2_2800305 | C->T | ATTGCCCCCTTGCTAGGTTATC | AGACGATTGGATGGTTGTGAAAC | PCR failure |
| F10L2 | Chr2_3225533 | C->T | GTGTTATCCTTTCCGGTTTCAG | GTTTAATCCCTTGGAGGTTGAG | Confirmed |
| F10L2 | Chr2_13971245 | G->A | AGGGTTCAGGTTTACAACTAGG | CCTGTTGTCTATTTTATTTCCTT | Confirmed |
| F10L2 | Chr3_8212836 | Del (22 bp) | CTTAATCTCATCAACGACACA | AAAACATAACAAGCATCTTACC | Confirmed |
| F10L2 | Chr3_11094358 | Ins (1 bp) | GCCATAGTGTCATTATGCTTATGTG | CTTAACTAAAAGTCTAAGGATCCGA | Confirmed |
| F10L2 | Chr3_11638846 | C->T | TATAGCTCGTAGATGGGAGGAT | GCAACATTGAAACCGCAAA | Confirmed |
| F10L2 | Chr4_2844088 | G->A | TTTAGGGTTGATTCGATTGG | GAACGACGTGGTTGTCAAAT | Confirmed |
| F10L2 | Chr4_3151045 | C->T | AAAAGGAGAAATCGCAAGAG | AAATCCGTAAGTAAAGAGTGG | Confirmed |
| F10L2 | Chr4_7341497 | C->A | GGAAGATGAAGAAGAAGGCAC | CGTTGCTAACTCCACGATGT | Confirmed |
| F11L2 | Chr4_1949803 | G->A | CTCCTCACCGACTACTCTACC | CGACAGGGATTACTGTTTCTA | Confirmed |
| F10L2 | Chr4_18055621 | Del (12 bp) | TTCTCACTACCCATCTCCCT | TCTAAATTCAACCATGTGGC | Confirmed |
| F10L2 | Chr5_19427454 | T->C | CGTAGGCGATTAGAGTAAGG | TTTGCACGATGAGATGTATG | Confirmed |
| F10L2 | Chr5_20838995 | T->A | TATCTTTCTCACTTTGCCATCT | CCCTCCCTCTTCTTTGTAGC | Confirmed |
| F10L3 | Chr1_5499962 | G->A | GCTTACTTTAGCAAGGGATAC | CAACGGGTGCCAGATTAGAT | Confirmed |
| F10L3 | Chr1_14056770 | T->C | GTCTTCCAGGTTATTGTCGA | ATGCCAAGTTACTAAGGGTG | Confirmed |
| F11L3 | Chr1_15432695 | C->T | CTTGCCATCGGGTATGTAGT | GTGATTTGGAGCTGTTGACG | Confirmed |
| F10L3 | Chr1_15607028 | Del (1 bp) | GACTTTTCTTTGCTAGATGAGCC | CCTCTTTGCCTTCTGTGGTT | Confirmed |
| F10L3 | Chr2_1135101 | C->T | AGACATCGTCTAGCCCACAA | AGAGTCCATAGCCCAAGTGA | Confirmed |
| F10L3 | Chr2_6049268 | G->T | CTAAGGTGCTACGTGCGAGAT | AAACGGCGACAGTGGGTC | Confirmed |
| F10L3 | Chr2_11097454 | Del (1 bp) | TCAATAAGGAGTAATTCTTTCTGCC | ATATTATGGTTGAAATCTGCTTGG | Confirmed |
| F10L3 | Chr2_12387869 | Del (1 bp) | ATTTTCTATTTGCTGCTTGT | CTGTTGTTATACGATTCATT | Confirmed |
| F10L3 | Chr2_16919679 | Del (1 bp) | AATCAGGAGGATACTCCCAAGC | CATTCAGCTTTCACCACGAA | Confirmed |
| F10L3 | Chr3_5942504 | A->T | TGAGAAGCCATTGACCATTA | AGCCTCTACTGTTTCTTCTACTCC | Confirmed |
| F10L3 | Chr3_15666533 | T->A | CATCGGTTAGCTGATCCG | CATACCAAGATACCATAACAATA | Confirmed |
| F10L3 | Chr3_16077875 | Del (22 bp) | AGAGGTATGGGAACGGGTTTA | ATACCGCTAACAACACCATC | Confirmed |
| F10L3 | Chr3_16672443 | C->T | CCTAACGGCCTTCTCACATC | AATAACGGAGTCAACTTTCC | Confirmed |
| F10L3 | Chr4_5545183 | Del (1 bp) | TGTTCGAGTAATAGAGTTGCAG | CGATTAAACCGATAATCCTACC | Confirmed |
| F10L3 | Chr4_812950 | Del (1 bp) | CACTGAGAAGGATCAATAGGGCAT | CGTCGTTTGTGTTTTCTGTGTGAT | Confirmed |
| F10L3 | Chr4_13277334 | G->T | TTGTTTTCTTGCTTGGAC | CCCGACCACATAACAACACTAAC | Confirmed |
| F10L3 | Chr5_3275501 | T->A | CGAGAATATGAACCGCAAGT | TAGCTGGTCTTAGTAGTAGTTCCA | Confirmed |
| F10L3 | Chr5_4812341 | C->A | CTAGAAATACGATGTTGGTTAA | ATCACAGTGGCAAAGTGG | Confirmed |
| F10L3 | Chr5_17598182 | C->T | GTCGCTGGACACTGACACG | GGTTTAGACTTGGACCCGATG | Confirmed |
| F10L4 | Chr1_1560460 | A->G | ATTCGACAAACGATAACTGGG | ATCCGAACTAGAACTTAGAAAT | Confirmed |
| F10L4 | Chr1_1576361 | G->T | TATGTCCTTGAGATTTCGAGC | TTGGGTCAACCTTGGCTT | Confirmed |
| F10L4 | Chr1_10307932 | Del (9 bp) | AACGGCAACGTGATTGGTC | AACACAACTTACAATCCCTTTT | Confirmed |
| F10L4 | Chr1_20544609 | G->C | GACAAAGGTGGGCATACATC | GGTCTAATGGAGGTGGCTTC | Confirmed |
| F10L4 | Chr1_29665186 | G->A | TATTTAGCACGACCGAGTTG | CTGACGGAAGCATGTCTTGG | Confirmed |
| F10L4 | Chr2_945764 | Del (18 bp) | ACCAAACTAAGCCAGCAATT | TGGCAGAATCTTCGGTGAAT | Confirmed |
| F10L4 | Chr2_1998090 | C->T | TTGGAGACGAAGCGTTTGAT | TACCTCCTGTGAATAAACACCAAT | Confirmed |
| F10L4 | Chr2_17437659 | A->G | TGAGCACAAGTCCTCGTAGC | TAGACGGTAAGACAATCAACA | Confirmed |
| F10L4 | Chr3_5779905 | G->A | CTTTACGAGAACGAGAAGCAC | GATACCCATCCACCAACTCT | Confirmed |
| F10L4 | Chr3_10500038 | T->A | AAGGCACGAAGAGGTCAATA | GATTGATTCCCGATCCCAGT | Confirmed |
| F10L4 | Chr3_12174170 | C->T | CCATTTACAGCCCACAAGAT | CCTCATCTCAAGCCCAACGA | Confirmed |
| F10L4 | Chr3_18747765 | C->T | CATGTTTCCATGAAATTAACC | TTTTGTTTATGGGCCTTGC | Confirmed |
| F10L4 | Chr4_11446995 | A->T | AGGTCATCACGAACCCTC | GGGTTTATCTTGTACTAACTGAC | Confirmed |
| F10L4 | Chr5_2165591 | A->T | TTCATCAGCAGTACCCACG | CTGGTGCTGTGACTCTTGAC | Confirmed |
| F10L4 | Chr5_10083527 | Del (1 bp) | CGACGCTTCAGTGACCAG | CAGAAATTCAGAAAGGCATA | Confirmed |
| F10L4 | Chr5_13772087 | Del (3 bp) | TCCGCCAAGAAACGAGAT | AATTCCACGATACATTATGCTA | Confirmed |
| F10L4 | Chr5_17063924 | A->G | TATTGTGGTCTCGGTTTCC | AACAAGTTAAGGCCCAATA | Confirmed |
| F10L5 | Chr1_12299829 | T->A | AAGTGGAACAAGAATCGGTAA | GATTGACCAGCCAAATCC | Confirmed |
| F10L5 | Chr1_12299830 | A->T | AAGTGGAACAAGAATCGGTAA | GATTGACCAGCCAAATCC | Confirmed |
| F10L5 | Chr1_21480740 | Del (3 bp) | AGTTTCACCCGGTCCACA | TTTAGGTCGGCGCTCTTG | Confirmed |
| F10L5 | Chr1_25387621 | A->C | AGATATTAACAAACGGCCAAAC | AGCATACGCACGAGCATT | Confirmed |
| F11L5 | Chr1_25718399 | A->G | TCAAGTGTATAGGGTTTTCCGCA | TTGTTGATGCTTGCACATCTCG | PCR failure |
| F10L5 | Chr2_602407 | C->T | CATAAATCCGTTTTATTTTCTC | GTTCAAATTTCGAGTTCAGAG | Confirmed |
| F10L5 | Chr2_4554916 | G->A | GAGAATCACAAATTATCATGGTC | GTTATTAACGTTTCGCTTGAA | Confirmed |
| F10L5 | Chr2_13881660 | Del (2 bp) | TCAGCCAAAGCAATAGACGAA | CTCTGCGGTTGACAAAGGAAT | Confirmed |
| F10L5 | Chr3_4333069 | C->T | GAAAGGAGGACTATTTTATGACTTC | CATGCTTTCCAGACGTTAGAG | Confirmed |
| F10L5 | Chr3_16705392 | C->G | AGATAAACGCTTATGTACTAAACAG | GGAGCCAATTTGAGGGAAGA | Confirmed |
| F10L5 | Chr5_2965228 | A->T | TTCTTGCTTTACACGCTTTG | GCATTCGGGCTTGTTGAG | Confirmed |

* Del refers to deletion; Ins refers to insertion

**Table S7.** Numbers and frequencies of transitions (ts) and transversions (tv) identified in MA lines and populations grown under Control, Heat, and Warming conditions.

| Sample | Ts | | | | Tv | | | | Ts/Tv ^e^ |
| --- | --- | --- | --- | --- | --- | --- | --- | --- | --- |
|  | Total^a^ | Mean^b^ | MF^c^ | SEM^d^ | Total^a^ | Mean^b^ | MF^c^ | SEM^d^ |  |
| D10 | 23 | 4.6 | 0.46 | 0.06 | 8 | 1.6 | 0.16 | 0.06 | 2.88 |
| E10 | 42 | 8.4 | 0.84 | 0.10 | 27 | 5.4 | 0.54 | 0.09 | 1.56 |
| F10 | 34 | 6.8 | 0.68 | 0.10 | 20 | 4.0 | 0.40 | 0.08 | 1.70 |
| A16 | 43 | 8.6 | 0.54 | 0.08 | 17 | 3.4 | 0.21 | 0.03 | 2.52 |
| B22 | 84 | 16.8 | 0.76 | 0.04 | 46 | 9.2 | 0.42 | 0.03 | 1.83 |
| C19 | 76 | 15.2 | 0.8 | 0.06 | 12 | 2.4 | 0.12 | 0.03 | 6.33 |

^a^ Total number of transition or transversion; ^b^ Average number of transition or transversion; ^c^ MF of transition or transversion (per genome per generation); ^d^ Standard error of means

**Table S8.** Comparison of mutation frequency among different genic regions calculated from Control, Heat, and Warming MA lines (**A**) and MA populations (**B**).

**A**

| Mutation position |  | D10 | | |  | E10 | | | |  | | F10 | | | | |
| --- | --- | --- | --- | --- | --- | --- | --- | --- | --- | --- | --- | --- | --- | --- | --- | --- |
|  |  | Mean^a^ | MF^b^ | SEM^c^ |  | Mean | MF | SEM |  | | Mean | | | MF | SEM | |
| Synonymous |  | 0.6 | 0.06 | 0.02 |  | 0.8 | 0.08 | 0.04 | |  | | 0.6 | 0.06 | | | 0.02 |
| Nonsynonymous |  | 0.2 | 0.02 | 0.02 |  | 2.6 | 0.26 | 0.04 | |  | | 1.8 | 0.18 | | | 0.09 |
| Stopgain |  | 0.0 | 0.00 | 0.00 |  | 0.2 | 0.02 | 0.02 | |  | | 0.0 | 0.00 | | | 0.00 |
| Frameshift |  | 0.0 | 0.00 | 0.00 |  | 0.6 | 0.06 | 0.04 | |  | | 0.4 | 0.04 | | | 0.04 |
| Nonframeshift |  | 0.2 | 0.02 | 0.02 |  | 0.0 | 0.00 | 0.00 | |  | | 0.6 | 0.06 | | | 0.04 |
| Intron |  | 1.2 | 0.12 | 0.04 |  | 2.0 | 0.20 | 0.07 | |  | | 1.2 | 0.12 | | | 0.06 |
| Splicing |  | 0.0 | 0.00 | 0.00 |  | 0.0 | 0.00 | 0.00 | |  | | 0.2 | 0.02 | | | 0.02 |
| UTR |  | 0.2 | 0.02 | 0.02 |  | 1.6 | 0.16 | 0.09 | |  | | 1.0 | 0.10 | | | 0.03 |

**B**

| Mutation position |  | A16 | | |  | B22 | | | |  | | C19 | | | | |
| --- | --- | --- | --- | --- | --- | --- | --- | --- | --- | --- | --- | --- | --- | --- | --- | --- |
|  |  | Mean^a^ | MF^b^ | SEM^c^ |  | Mean | MF | SEM |  | | Mean | | | MF | SEM | |
| Synonymous |  | 1 | 0.06 | 0.02 |  | 0.2 | 0.01 | 0.01 | |  | | 1.6 | 0.08 | | | 0.03 |
| Nonsynonymous |  | 0.4 | 0.03 | 0.02 |  | 1.6 | 0.07 | 0.01 | |  | | 3.4 | 0.18 | | | 0.01 |
| Stopgain |  | 0.0 | 0.00 | 0.00 |  | 0.2 | 0.01 | 0.01 | |  | | 0.0 | 0.00 | | | 0.00 |
| Frameshift |  | 0.2 | 0.01 | 0.01 |  | 0.0 | 0.00 | 0.00 | |  | | 0.0 | 0.00 | | | 0.00 |
| Nonframeshift |  | 0.0 | 0.00 | 0.00 |  | 0.8 | 0.04 | 0.01 | |  | | 0.0 | 0.00 | | | 0.00 |
| Intron |  | 3.4 | 0.21 | 0.02 |  | 5.2 | 0.24 | 0.01 | |  | | 3.4 | 0.18 | | | 0.03 |
| Splicing |  | 0.2 | 0.01 | 0.01 |  | 0.0 | 0.00 | 0.00 | |  | | 0.0 | 0.00 | | | 0.00 |
| UTR |  | 0.6 | 0.04 | 0.02 |  | 3.4 | 0.16 | 0.02 | |  | | 0.0 | 0.00 | | | 0.00 |

^a^ Mean number of mutations in each treatment; ^b^ Mutation frequency (per genome per generation); ^c^ Standard errors of the means

**Table S9.** Whole-genome bisulfite sequencing and the numbers of methylated cytosine positions in genomic DNA samples.

| Sample | All clean reads | Mapped reads | BS conversion rate(%) | Number of total mC (%) | Number of mCpG (%) | Number of mCHG (%) | Number of mCHH (%) |
| --- | --- | --- | --- | --- | --- | --- | --- |
| D10L1 | 31936560 | 25865419 | 99.39% | 4183314 (9.71%) | 1525431 (27.22%) | 891548 (14.55%) | 1766335 (5.63%) |
| D10L2 | 29577108 | 23892387 | 99.12% | 4185456 (9.72%) | 1594261 (28.45%) | 879630 (14.35%) | 1711565 (5.46%) |
| D10L3 | 29444399 | 23991296 | 98.70% | 4192174 (9.73%) | 1579003 (28.17%) | 894014 (14.59%) | 1719157 (5.48%) |
| D10L4 | 29052699 | 23645991 | 98.91% | 4256645 (9.88%) | 1564243 (27.91%) | 896055 (14.62%) | 1796347 (5.73%) |
| D10L5 | 30802521 | 24672819 | 99.50% | 4262303 (9.89%) | 1555523 (27.76%) | 881253 (14.38%) | 1825527 (5.82%) |
| E10L1 | 30230292 | 24867438 | 99.49% | 4458137 (10.35%) | 1563104 (27.89%) | 933699 (15.24%) | 1961334 (6.26%) |
| E10L2 | 29804480 | 24079039 | 99.50% | 4427955 (10.28%) | 1546435 (27.59%) | 907644 (14.81%) | 1973876 (6.3%) |
| E10L3 | 36431078 | 29636681 | 99.43% | 4608944 (10.7%) | 1611142 (28.75%) | 951590 (15.53%) | 2046212 (6.53%) |
| E10L4 | 30070173 | 24702647 | 99.10% | 4551692 (10.57%) | 1506854 (26.89%) | 914128 (14.92%) | 2130710 (6.8%) |
| E10L5 | 30104442 | 24336430 | 99.63% | 4652749 (10.8%) | 1542426 (27.52%) | 924482 (15.08%) | 2185841 (6.97%) |
| F10L1 | 28024618 | 22711150 | 99.42% | 4360821 (10.12%) | 1485592 (26.51%) | 907678 (14.81%) | 1967551 (6.28%) |
| F10L2 | 29909129 | 24127694 | 99.43% | 4274168 (9.92%) | 1557088 (27.78%) | 897459 (14.64%) | 1819621 (5.8%) |
| F10L3 | 32368538 | 26574569 | 99.58% | 4778645 (11.09%) | 1535721 (27.4%) | 957539 (15.62%) | 2285385 (7.29%) |
| F10L4 | 33764819 | 27541962 | 99.14% | 4425482 (10.27%) | 1615081 (28.82%) | 916592 (14.96%) | 1893809 (6.04%) |
| F10L5 | 27123602 | 21717868 | 99.61% | 4650640 (10.8%) | 1537691 (27.44%) | 946337 (15.44%) | 2166612 (6.91%) |

**Table S10.** Logistic regression analysis of the effects of cytosine methylation and TE regions on the likelihood of a given nucleotide being mutated in the Control, Heat, and Warming MA lines.

|  |  | *p*-value |  |
| --- | --- | --- | --- |
| **Control D** |  |  |  |
| Main effect | TE | 0.007 | ** |
|  | Methylated | 0.005 | ** |
| Two-way interaction | TE: Methylated | 0.450 |  |
| **Heat E** |  |  |  |
| Main effect | TE | 0.041 | * |
|  | Methylated | 4.77E-05 | *** |
| Two-way interaction | TE: Methylated | 0.199 |  |
| **Warming F** |  |  |  |
| Main effect | TE | 0.011 | * |
|  | Methylated | 1.46e-06 | *** |
| Two-way interaction | TE: Methylated | 0.132 |  |

*p*-value < 0.01 (**) and < 0.001 (***) are indicated.

**Table S11.** Genomic locations and affected bases of indels under Control, Heat, and Warming conditions.

| Group | Sample ID | Chrm | Position | | Type | Ref seq | Alt seq | Upstream seq | Downstream seq |
| --- | --- | --- | --- | --- | --- | --- | --- | --- | --- |
| Control | D10 | Chr1* | 8500098 | 8500098 | Ins(1) | C | CA | TACCGTTGAA | AAAAAAAAACAC |
|  |  | Chr2* | 9481937 | 9481943 | Del(6) | ATTGAGT | A | TCAGCGAAC | GGTCCGGTGCT |
|  |  | Chr2* | 13702917 | 13702918 | Del(1) | CT | C | AATTTACTCT | TTTTTTTCATAA |
|  |  | Chr2* | 17323097 | 17323097 | Ins(1) | C | CT | CATGCAGCCA | TTTCAAGCAGAAAAAG |
|  |  | Chr3* | 3299183 | 3299184 | Del(1) | TA | T | AAAATACATGAC | AAAAAAAAAGT |
|  |  | Chr3* | 6651225 | 6651225 | Ins(2) | C | CCT | TATTTTTCTAATT | CTCTCTCTCTCTCTCTC |
|  |  | Chr4** | 388431 | 388431 | Ins(3) | G | GCTT | TCTCCTACACTC | CTTCTTCTTCTTCTTC |
|  |  | Chr5* | 8088477 | 8088478 | Del(1) | CT | C | CGGCAATCA | TTTGAATCGA |
|  | A16 | Chr3 | 7976019 | 7976019 | Ins(1) | C | CT | GCAAACCCCAA | TTGCTCGTTATCTC |
|  |  | Chr3* | 12506063 | 12506064 | Del(1) | TA | T | AAAAAATTCA | AAAAACAAGATC |
|  |  | Chr3* | 18672292 | 18672292 | Ins(1) | T | TA | TTCAACCGGCCA | AAAAAAGTTTGTTCTC |
|  |  | Chr4* | 10705601 | 10705602 | Del(1) | AT | A | AAAAAAAAA | ATATATATATAT |
|  |  | Chr4* | 14381494 | 14381494 | Ins(1) | T | TA | CTGGAGTACT | AAAAGCTGTA |
|  |  | Chr5* | 20525980 | 20525981 | Del(1) | AT | A | TATATCTCG | TCGGCACTGG |
| Heat | E10 | Chr1* | 1933381 | 1933382 | Del(1) | CA | C | TTTAAGTCACC | AAAAAAAAAAAAAAAT |
|  |  | Chr1* | 11957864 | 11957865 | Del(1) | CT | C | TGTCTTCCAGAG | TTTTTTTTTTTCTGTAAA |
|  |  | Chr1* | 16140729 | 16140730 | Del(1) | GT | G | TTATTCCTCT | TTTTTTCAGAATTTT |
|  |  | Chr1** | 18097746 | 18097750 | Del(4) | TTTTC | T | GTCATATGT | TTTCTTTCTAT |
|  |  | Chr1* | 18941042 | 18941043 | Del(1) | CA | C | GTAACCAAAC | AAAAAAAAAGGTC |
|  |  | Chr1* | 26847184 | 26847185 | Del(1) | CA | C | TTTCCTCTG | AAAGCAATAG |
|  |  | Chr2* | 1655746 | 1655748 | Del(2) | CTA | C | GAGTAGAAAG | TATATATATATATA |
|  |  | Chr2** | 1761183 | 1761186 | Del(3) | TAGA | T | GGATAAGAAG | AGAAGAAGAAGAAG |
|  |  | Chr2* | 8060293 | 8060293 | Ins(1) | T | TG | GGAACTAGCT | GGGGGTTAGAGTTAAG |
|  |  | Chr2* | 12003172 | 12003173 | Del(1) | AT | A | CTAGAGGCT | ATGTGAAAAAAAA |
|  |  | Chr3* | 234615 | 234616 | Del(1) | CT | C | TCATCAACCT | TTTTTACTGCTCTTG |
|  |  | Chr3* | 10719892 | 10719893 | Del(1) | AT | A | CTTTCTCATTT | TTTTTTTTACGTTGA |
|  |  | Chr3* | 13525198 | 13525199 | Del(1) | GA | G | CAAATAGAA | AAAAAAAAAATGGTT |
|  |  | Chr3* | 17912371 | 17912372 | Del(1) | TA | T | TTTATGGAAT | AAAAAAAAAAGTGTA |
|  |  | Chr3* | 19139287 | 19139288 | Del(1) | AT | A | ATTTATTGC | TGCTTTCTCTTCTCTT |
|  |  | Chr4* | 1507947 | 1507947 | Ins(2) | C | CAT | AAAATCTCTA | ATATATATATATCTATG |
|  |  | Chr4* | 11591111 | 11591112 | Del(1) | TA | T | GAAACCCCCT | AAAAAATGAGAAAAC |
|  |  | Chr4 | 14602865 | 14602866 | Del(1) | CG | C | CATGGTCGA | TACGCCATTT |
|  |  | Chr4* | 17786592 | 17786593 | Del(1) | GA | G | GATGAGAAG | AAAGGTACAA |
|  |  | Chr4* | 18096195 | 18096197 | Del(2) | TGA | T | ACAGACACCAG | GAGAGAGAGAGAGAGAG |
|  |  | Chr5* | 1383660 | 1383661 | Del(1) | AT | A | TATTTTCTTC | TTTTTTTTTTTTTTG |
|  |  | Chr5* | 7909461 | 7909463 | Del(2) | GGA | G | GAGAGGTGGC | GAGAGAGAGAGAGAA |
|  |  | Chr5* | 11310913 | 11310914 | Del(1) | AT | A | GGTACATCT | TTTTTTTTTTCTCCA |
|  |  | Chr5* | 12089325 | 12089326 | Del(1) | AT | A | TCCCATAAAT | TTTTTTGGTATATTT |
|  |  | Chr5* | 13526118 | 13526118 | Ins(1) | A | AT | TCAATTGTTG | TTTTTTTTTTTTGGGT |
|  |  | Chr5* | 18040164 | 18040165 | Del(1) | CA | C | TCACACATGA | AAAAAAATAAAAATT |
|  |  | Chr5* | 19774482 | 19774483 | Del(1) | TG | T | AATGTCATC | GGATGCTTTC |
|  |  | Chr5* | 21660950 | 21660951 | Del(1) | GA | G | ATAAAACTC | AACTAGGGAG |
|  | B22 | Chr1* | 12736204 | 12736204 | Ins(1) | C | CA | TTACACAAACCA | AAAAAAAAAAATCC |
|  |  | Chr1* | 17211130 | 17211131 | Del(1) | CA | C | ATTCACAAAAA | AAAAAAAAAAGTTAAG |
|  |  | Chr1* | 17296297 | 17296298 | Del(1) | GA | G | ATTCTAGTGAA | AAAAAAAAATATTGTT |
|  |  | Chr1* | 21055168 | 21055169 | Del(1) | AT | A | AAAAAAACACT | TTTTTTTTGCTCTTTGAT |
|  |  | Chr2* | 8221769 | 8221771 | Ins(2) | TTC | T | AGAAGCTCGCT | TCTCTCTCTCTCTCGTG |
|  |  | Chr3* | 2609727 | 2609728 | Del(1) | AC | A | CAACAAACA | CCCCAATCATA |
|  |  | Chr3* | 12426325 | 12426326 | Del(1) | TA | T | AATAAAAGA | AAAAAAAAAATAACAT |
|  |  | Chr3 | 22521170 | 22521170 | Ins(17) | A | AGGTGATTTTGCAGTTTT | TATGGCTCCCG | GGTGATTTTGCAGTTTTGGCGGCGG |
|  |  | Chr3* | 22931095 | 22931096 | Del(1) | CT | C | AGTAATGTCTCT | TTTTTTTTTATTTTTTTT |
|  |  | Chr4* | 8669182 | 8669183 | Del(1) | TA | T | CGCTAGGTTGT | AAAAAAAAAAGTTA |
|  |  | Chr4** | 9870036 | 9870039 | Del(3) | GTGT | G | TGTCTACCATTT | TGTTGTTGTTGTTGTT |
|  |  | Chr4** | 16895578 | 16895578 | Ins(4) | C | CTATA | TCTAAAAGGC | TATATATATATA |
|  |  | Chr4* | 17737645 | 17737646 | Del(1) | AT | A | AAATCTGAAAAC | TTTTTTTTTTCTTGAATC |
|  |  | Chr5* | 7356476 | 7356477 | Del(1) | GT | G | AAACATTTTTTT | TTTTTTTTTGTTTTTATC |
|  |  | Chr5* | 13976746 | 13976747 | Del(1) | AT | A | AAATGTTTTAA | TTTTTTTTATTATCTATA |
|  |  | Chr5* | 15578648 | 15578648 | Del(1) | TA | T | CACAATTTACA | AAAAAAAAAATAACAATT |
|  |  | Chr5* | 23370350 | 23370350 | Ins(2) | C | CTT | TTAGTTTGGAAC | TTTTTTTTTTTAAGTTAGG |
| Warming | F10 | Chr1* | 6677325 | 6677325 | Ins(1) | C | CA | TTAAAAAATC | AAAAAAAAAAGAGAGAA |
|  |  | Chr1** | 10307931 | 10307940 | Del(9) | CTAGTAGTAG | C | GTTGAGCTG | TAGTAGTAGTAGTAGTAG |
|  |  | Chr1* | 15607027 | 15607028 | Del(1) | CT | C | AATGAAGAT | TTTATTTGGTTT |
|  |  | Chr1 | 21480739 | 21480742 | Del(3) | ATCT | A | TATAACAGC | GCATTATTTAAAAG |
|  |  | Chr1* | 26011709 | 26011710 | Del(1) | AC | A | AGAGATGGG | GATTGCCAAAAAAGGG |
|  |  | Chr2** | 945763 | 945781 | Del(18) | TGATGAGAAGAATCTCAAG | T | TGAGGTAAG | GATGAGAAGAATCTCAAG |
|  |  | Chr2* | 11097453 | 11097454 | Del(1) | TA | T | TGGTATATAT | AAAAAAAATTATAAT |
|  |  | Chr2* | 12387868 | 12387869 | Del(1) | TC | T | GTTAACAAT | AGTAACTCACGT |
|  |  | Chr2* | 13881659 | 13881661 | Del(2) | GGA | G | AGAATGCTT | AAGTATGCAA |
|  |  | Chr2* | 16919678 | 16919679 | Del(1) | AG | A | ACGTCTTTA | GATGTTTGTCTCTGTT |
|  |  | Chr3 | 8212835 | 8212857 | Del(22) | GGTTTGAAAACAGCTATCGTGCT | G | TCTTCGACT | GTAAAATGTAAAGCA |
|  |  | Chr3* | 11094358 | 11094358 | Ins(1) | G | GA | AGAACAAACA | AAAAAAAAATGATAAA |
|  |  | Chr3** | 16077874 | 16077896 | Del(22) | AAAACATTGAATAATGTTTACCT | A | AAATATCTA | AAACATTGAATAATGTTTACCTAAA |
|  |  | Chr4* | 812949 | 812950 | Del(1) | CT | C | CCTAAAAAGGG | TTTTTTGGCCCATTA |
|  |  | Chr4* | 5545182 | 5545183 | Del(1) | GT | G | ACCCTAATAAA | TTTTCCCCAAATTGTTTT |
|  |  | Chr4** | 18055620 | 18055632 | Del(12) | GCATACATACATA | G | TCTACATCT | CATACATACATACATACATACATA |
|  |  | Chr5* | 5229726 | 5229726 | Ins(3) | C | CCTT | ATATCCCCTTAC | CTTCTTCTTCTTCTTCTT |
|  |  | Chr5* | 10083526 | 10083527 | Del(1) | CT | C | TGATCTACT | TTTTATGGTT |
|  |  | Chr5** | 13772086 | 13772089 | Del(3) | AAAT | A | TAAAACTAT | AATTAGGACA |
|  |  | Chr5* | 24779065 | 24779067 | Del(2) | CTT | C | ATGTAAAAC | TTTATATATCCGAGG |
|  | C19 | Chr1 | 12248543 | 12248544 | Del(1) | CA | C | TTTTAAAATAA | AAAAAAAAAATAAAATAA |
|  |  | Chr1 | 22878972 | 22878972 | Ins(2) | A | ATG | AGTAATTTATAT | TGTGTGTGTGTGTATGTTG |
|  |  | Chr2 | 5521440 | 5521440 | Ins(1) | T | TC | GCATCTTCAATC | CCCCCCCCCCTTTTATCTT |
|  |  | Chr2 | 9853582 | 9853582 | Ins(2) | T | TTA | ACATCTTTTATC | TATATATATATAGCCCCTA |
|  |  | Chr3 | 391260 | 391261 | Del(1) | GT | G | AAACAGTACAC | TTTTTTTTTTGTTTCTTC |
|  |  | Chr3 | 17999629 | 17999630 | Del(1) | AT | A | TCTATGTTCGAT | TTTTTTTTTATGAACGTG |
|  |  | Chr4* | 5357832 | 5357833 | Del(1) | TA | T | GTTATTGCA | AACTACTATCA |
|  |  | Chr4* | 5592844 | 5592884 | Del(40) | CTGATTTTGTATGGAACTCATAGTGAGTTTAATGTGATCAT | C | AGTGAAAAA | TGATTCAGGAG |
|  |  | Chr4** | 7807195 | 7807195 | Ins(3) | C | CAGA | TTTGTATAATTA | AGAAGAAGAAGAAGAA |
|  |  | Chr4* | 6059380 | 6059381 | Del(1) | GT | G | AAAGAAACA | AAAAAGAGAAA |
|  |  | Chr5* | 3815832 | 3815832 | Ins(2) | T | TTA | TTTCTAAAACGG | TATATATATATACCAACGA |
|  |  | Chr5* | 15790537 | 15790538 | Del(1) | CA | C | AGACCATTTTC | AAAAAAAATTCAACCATC |
|  |  | Chr5* | 25824648 | 25824648 | Del(1) | GT | G | CTTGGTGTGT | TTTTTCTTCAAAAATATT |

* The indel occurred near simple repeats. ** The indel involve tandem repeat sequences.
