# Supplementary material for: Genome-wide DNA mutations in Arabidopsis plants after multigenerational exposure to high temperatures
Source: Genome Biol. 2021 May 25;22:160. doi: 10.1186/s13059-021-02381-4 (PMC8145854; doi:10.1186/s13059-021-02381-4)
Supplement: Supplementary file 2 — Additional file 2: Fig. S1. KEGG enrichment of mutated genes in the Control (A and D), Heat (B and E), and Warming (C and F) treatments. Fig. S2. Mutation rates estimated based on the interactions between TEs/non-TEs and methylation/unmethylation sites in the Control D (A), Heat E (B) and Warming F (C) MA lines. The interactions were classified as follows: non-TE with unmethylated sites, non-TEs with methylated sites, TEs with unmethylated sites, and TEs with methylated sites. In each case, the mutation rate was calculated by dividing the number of observed mutations by the number of analyzed sites capable of producing a given mutation, and the number of generations of MA in each Control, Warming, and Heat line. Differences in mutation rates were evaluated using Student’s t-test. Asterisks indicate significant differences from Control D at p < 0.05 (*). Fig. S3. Relative mutation rates by local GC content across the A. thaliana genome of MA lines (Control D, Heat E, and Warming F) and populations (Control A, Heat B, and Warming C). A 1-kb bin size and 0.005 intervals of GC content were used (see Methods). The figure was plotted using the loess method and stat_smooth function of the ggplot2 R package). Relative mutation rate was calculated as described in Methods. [file 13059_2021_2381_MOESM2_ESM.docx]

**Supplementary Figures**


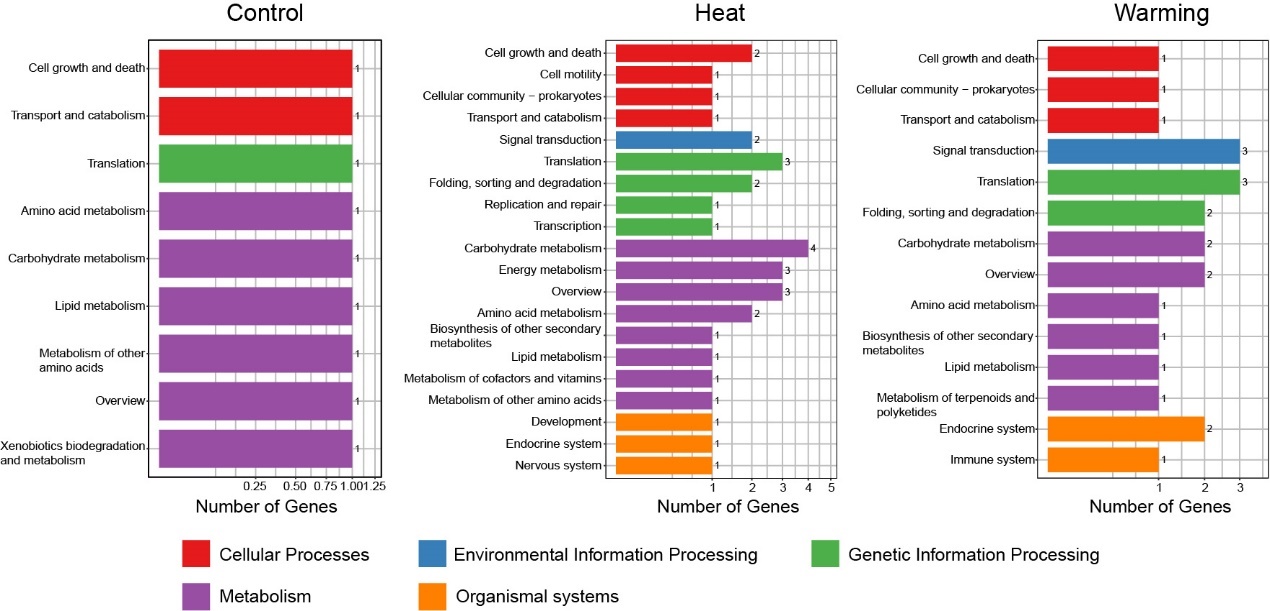


**Figure S1.** KEGG enrichment of mutated genes in the Control (A and D), Heat (B and E), and Warming (C and F) treatments.


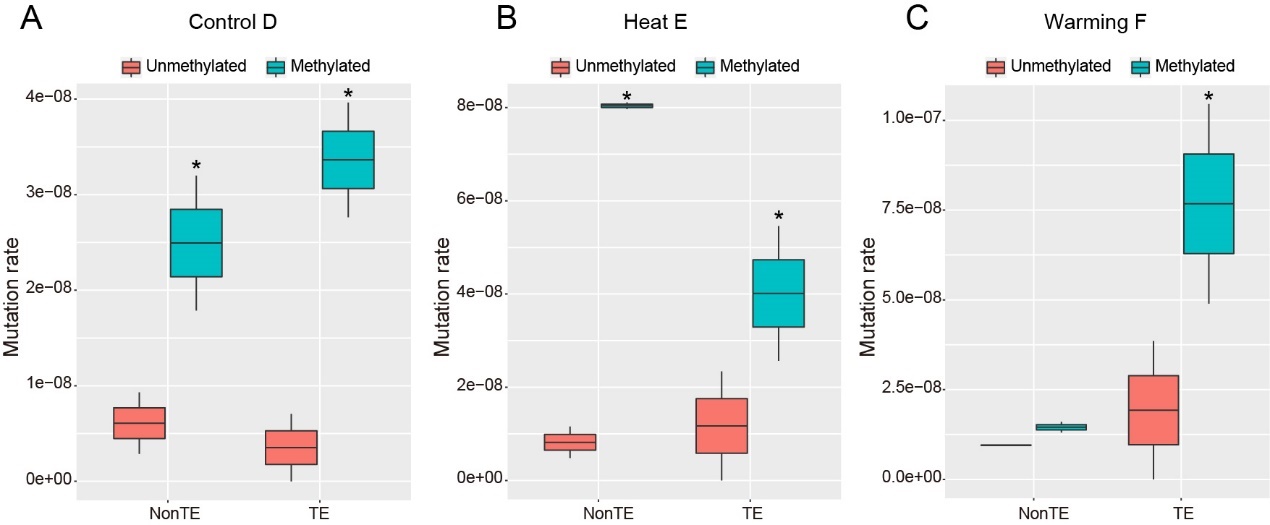


**Figure S2.** Mutation rates estimated based on the interactions between TEs/non-TEs and methylation/unmethylation sites in the Control D (A), Heat E (B) and Warming F (C) MA lines. The interactions were classified as follows: non-TE with unmethylated sites, non-TEs with methylated sites, TEs with unmethylated sites, and TEs with methylated sites. In each case, the mutation rate was calculated by dividing the number of observed mutations by the number of analyzed sites capable of producing a given mutation, and the number of generations of MA in each Control, Warming, and Heat line. Differences in mutation rates were evaluated using Student’s t-test. Asterisks indicate significant differences from Control D at p < 0.05 (*).


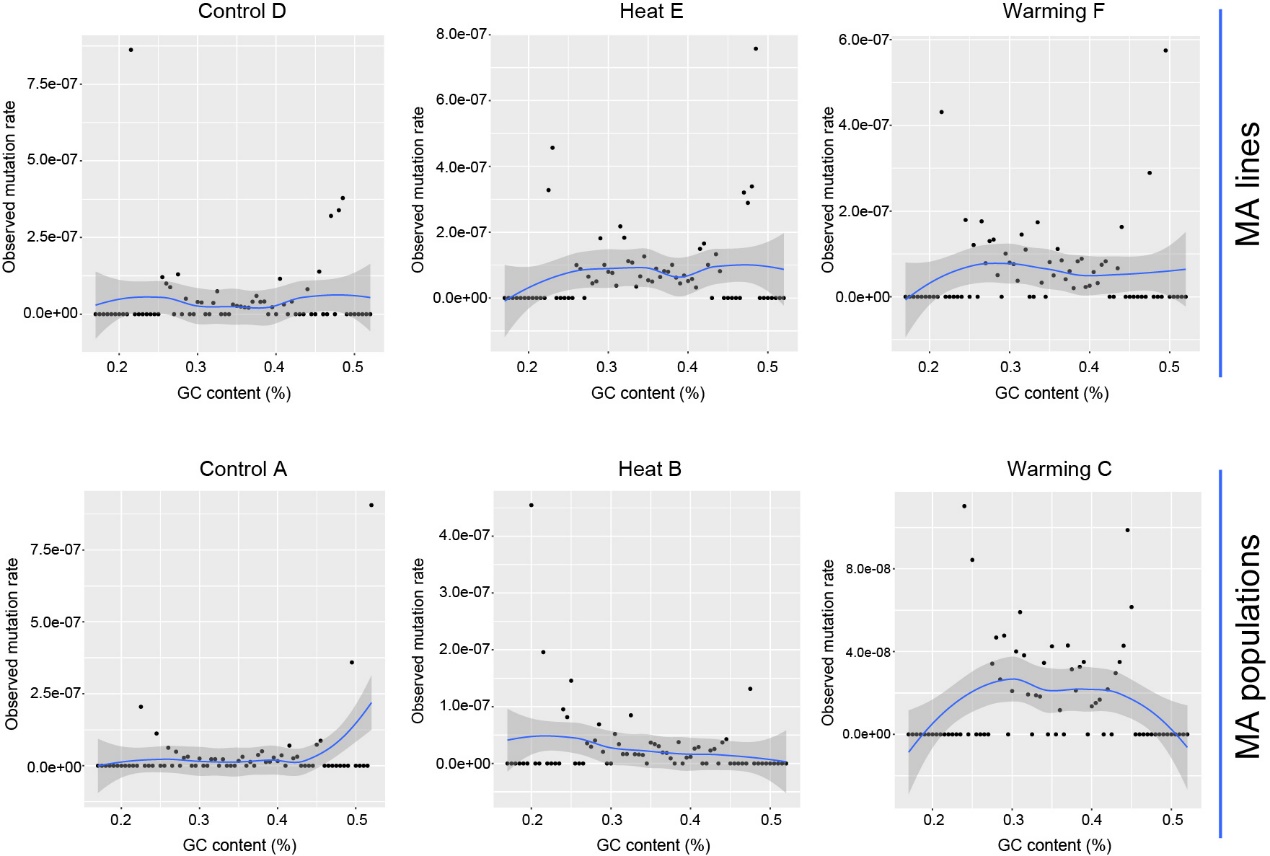


**Figure S3.** Relative mutation rates by local GC content across the *A. thaliana* genome of MA lines (Control D, Heat E, and Warming F) and populations (Control A, Heat B, and Warming C). A 1-kb bin size and 0.005 intervals of GC content were used. The figure was plotted using the loess method and stat_smooth function of the *ggplot2* R package. Relative mutation rates were calculated as described in the Methods.
