# Supplementary material for: Genome-wide DNA mutations in Arabidopsis plants after multigenerational exposure to high temperatures
Source: Genome Biol. 2021 May 25;22:160. doi: 10.1186/s13059-021-02381-4 (PMC8145854; doi:10.1186/s13059-021-02381-4)
Supplement: Supplementary file 3 — Additional file 3: Supplementary detailed methods. [file 13059_2021_2381_MOESM3_ESM.docx]

**Supplementary detailed methods**

**Temperature treatments for MA line and population experiments**

To investigate the accumulation of mutations in *A. thaliana* grown under different temperature conditions over successive generations, we designed three temperature treatments for MA lines (single-seed descent maintained under laboratory conditions) and populations (simulation experiments) in growth chambers: 23/18°C (light/dark), 28/23°C, and 32/27°C (one growth chamber per temperature regime) (Fig. 1a). The 23/18°C (ambient temperature) regime was used as the Control treatment (groups D and A), because most studies use 22–23°C as the baseline temperature to investigate the effects of temperature on *Arabidopsis* [1, 2]. The 28/23°C (elevated 5°C relative to Control) regime was used as the Warming treatment (groups F and C) to simulate global warming according to a report that the global mean temperature is likely to warm by 1.5–4°C by the end of this century [3]; this treatment also represents an intermediate temperature between the Control and Heat treatments. The 32/27°C (elevated 9°C relative to control) regime was used as the Heat treatment (groups E and B), which is the maximum temperature under which *A. thaliana* can propagate for multiple generations. Because plants grown under 32/27℃ have reached the upper limits for sexual reproduction, with greatly reduced seed-set rates, short siliques, fewer seeds, decreased leaf size (Fig. 1b–d), etc., plants grown at temperatures ≥ 33℃ would not produce enough seeds for the next generation.

Considering the gradual change in mean temperature seen in nature, for the Heat MA population (group B), we simulated a gradual warming through stepwise temperature increases in several preceding generations until the temperature reached 32℃. In detail, the temperature was elevated by 1°C per generation for the first nine generations [from 24/19°C (G1), to 25/20°C (G2), 26/21°C (G3), 27/22°C (G4), 28/23°C (G5), 29/24°C (G6), 30/25°C (G7), 31/26°C (G8), and finally 32/27°C (G9)], and then maintained at 32/27°C for the subsequent 13 generations (Fig. 1a). To ensure the survival of seedlings in Heat (32/27°C) treatments (groups E and B), after cotyledons fully opened (i.e., stage 1.0) [4], we increased the temperature from 28/23°C to 32/27°C over a span of 3–4 days, then maintained at the temperature at 32/27°C.

We conducted this study using three temperature regimes, with five replicates per regime at the line level and 30 individuals at the population level (five individuals were selected for the next generation). These lines and populations have been maintained through many generations in 15 growth chambers over 10 years. This study involved tremendous labor, facilities, and time; thus, we are unable to grow more lines or populations through similar multigenerational cultivation.

**For MA lines**, ~100 seeds from the ancestral plant were isolated after 2 d of stratification at 4°C, and about 75 germinated seedlings were sown and finally thinned to 15 seedlings to establish the single-seed line system (15 lines; one seedling per line) (Fig. 1a, b). The 15 lines were divided into three groups (five lines per group) for the Control, Warming, and Heat treatments. They were planted in sterilized substrate [1:1 (v/v) mixture of vermiculite and peat] in individual pots, and the growth conditions were consistent with those of their ancestral plant (described above), except for the temperature settings. The resultant “Generation 0 (G0)” (Fig. 1a) plants from the three groups were grown and self-pollinated under Control, Warming, and Heat conditions in six independent champers, respectively. To offset any possible effect of position within each chamber, and the effects of the different chambers, the pots were randomly rearranged within each chamber, and interchanged among chambers periodically. After G0 seeds were collected separately, five “Generation 1 (G1)” seedlings from each line within different temperature treatments were sown, while a single plant (after seedlings emerged) was randomly chosen to propagate the next generation. The remaining seeds from each line were stored in microcentrifuge tubes with desiccants. This process was repeated for nine subsequent generations under the Control, Warming, and Heat conditions, leading to 15 independent MA lines within the three treatments (five lines per treatment) spanning 10 successive generations.

**For MA populations**, ~300 seeds from the same ancestral plant were initially isolated and then divided into three groups (35 seedlings per group) for the Control, Warming, and Heat treatments, respectively. Each population (group) was propagated in each generation using mixed seeds from five individual vigorous plants chosen from among the 35 seedlings (Fig. 1a, b). Except for the temperature treatment of the B group (described above), all growth conditions and management procedures within the chambers were identical to those in the MA lines. The MA populations grown under the three different treatments were propagated for more successive generations than the MA lines, with 16 generations for Control (A), 22 generations for Heat (B), and 19 generations for Warming (C). The accumulated generations for each population treatment differed, because warmer temperatures resulted in shorter generation times; thus, more generations grew under the Heat and Warming conditions than under the Control conditions within the same time period.

To maximize coverage of the genetic background of the ancestral plant, ~100 seeds of the ancestral plant were sown to generate 100 individuals grown under the same conditions as the progenitor. Among these 100 individuals, five plants were randomly selected and sampled as the progenitors to establish the genetic background of the MA lines and populations.

At stage 5 (bolting) and stage 8–9 (silique ripening), randomly selected leaves (~30) and siliques (~30) from 35 plants per treatment (Control, Heat, and Warming) were photographed using a digital camera, and the leaf area and silique length were measured using ImageJ software (NIH, Bethesda, MD, USA). Three replicates from each treatment were analyzed.

**Mutation rate estimation**

The mutation frequency (per genome per generation) and rate (per site per generation) in each treatment were estimated by the formulas *m* = *n*/*g* and *μ* = *n*/*gb* (or *μ* = *m/b*). For example, among the single-seed descent MA lines, Heat E10L1 had 15 SNVs (*n* = 15) and *g* = 10; therefore, the mutation frequency *m* = 15/10 = 1.5 SNVs per generation per line. Similarly, the SNV frequencies of the Heat E10L2, E10L3, E10L4, and E10L5 lines were calculated as 1.4 (14/10), 1.6 (16/10), 1.4 (14/10), and 1.0 (10/10), respectively, and the average mutation frequency of Heat E was 1.4 (∑*m*/5) mutations per generation. Based on the calculated mutation frequency, we further estimated the per site per generation mutation rate using the formula *μ* = *n*/*gb* or (*m/b*), where *b* is the number of accessible reference sites analyzed. Taking Heat E10L1 as an example, the mutation rate *μ*_SNV_ = 1.5/ 117,293,204 (the reference sites accessible for variant calling) = 1.28e-08, and correspondingly, the final SNV mutation rate of Heat E calculated by averaging the individual mutation rates was *μ* = 1.18e-08.

However, due to the limited number of generations in our MA experiments, actual mutation rates may be underestimated using the above calculations [5, 6]. The reason for the potential underestimation of mutation rates has been analyzed in detail by [7]. In brief, the putative formula *gμ* + 1/2*τ* (*g* − 2 + (1/2)*^g^*^−1^) was used to show the total probability of accumulated homozygous mutations over *g* generations, where *μ* and *τ* represent the probabilities of a new homozygous and new heterozygous mutation per site per generation, respectively. As a consequence, after *g* generations, the count of mutations was *N* · *g* · (*μ* + 1/2*τ*), where *N* is the number of sites. In the present case, *g* = 10, and the mutation rate (per site per generation) obtained by dividing the count of identified homozygous mutations by the number of generations and the number of sites is *μ* + 4*τ*/10, which is lower than the expected homozygous mutation rate, that is, *μ* + 5*τ*/10. Because of the difficulty in estimating *μ* and *τ* during plant development [5, 6], we are not able to accurately correct the underestimation. However, this underestimation (20% underestimation of the actual mutation rate) had a limited effect on our results and conclusion, similar to analyses performed by [5] and [7]. Since we applied the same approach to estimate mutation rates in MA lines and populations under Control, Heat, and Warming conditions, the underestimation should have little effect when comparisons of mutation rates are performed.

**References**

1. Lee JH, Yoo SJ, Park SH, Hwang I, Lee JS, and Ahn JH. Role of *SVP* in the control of flowering time by ambient temperature in *Arabidopsis*. Genes Dev. 2007;21:397–402.
2. Jin B, Wang L, Wang J, Jiang KZ, Wang Y, Jiang XX, et al. The effect of experimental warming on leaf functional traits, leaf structure and leaf biochemistry in *Arabidopsis thaliana*. BMC Plant Biol. 2011;11:35.
3. Flato G, Marotzke J, Abiodun B, Braconnot P, Chou SC, Collins W, et al. Evaluation of climate models, in: *Climate Change 2013*: *The Physical Science Basis*. *Contribution of Working Group I to the Fifth Assessment Report of the Intergovernmental Panel on Climate Change*, edited by Stocker T.F., et al. Cambridge Univ. Press, Cambridge, U. K. and New York, USA, 2013. pp. 741–866.
4. Boyes DC, Zayed AM, Ascenzi R, McCaskill AJ, Hoffman NE, Davis KR, et al. Growth stage-based phenotypic analysis of Arabidopsis: a model for high throughput functional genomics in plants. Plant Cell. 2001;13:1499–510.
5. Ossowski S, Schneeberger K, Lucas-Lledó JI, Warthmann N, Clark RM, Shaw RG, et al. The rate and molecular spectrum of spontaneous mutations in *Arabidopsis thaliana*. Science. 2010;327:92–4.
6. Hoffman PD, Leonard JM, Lindberg GE, Bollmann SR, Hays JB. Rapid accumulation of mutations during seed-to-seed propagation of mismatch-repair-defective *Arabidopsis*. Genes Dev. 2004;18:2676–85.
7. Jiang C, Mithani A, Belfield EJ, Mott R, Hurst LD, Harberd NP. Environmentally responsive genome-wide accumulation of de novo *Arabidopsis thaliana* mutations and epimutations. Genome Res. 2014;24:1821–9.
